# Multimodal data integration to determine viral and innate immune kinetics in human airway epithelium

**DOI:** 10.1371/journal.pcbi.1014248

**Published:** 2026-05-20

**Authors:** Pascal Lukas, Aurélien Gibeaud, Clarisse Schumer, Jonas Arruda, Jeremie Guedj, Olivier Terrier, Frederik Graw

**Affiliations:** 1 Department of Internal Medicine 5, Haematology and Oncology, Friedrich-Alexander-Universität Erlangen-Nürnberg and Universitätsklinikum Erlangen, Erlangen, Germany; 2 CIRI, Centre International de Recherche en Infectiologie, Team VirPath, Université de Lyon, INSERM U1111, Université Claude Bernard Lyon 1, CNRS, UMR5308, ENS de Lyon, Lyon, France; 3 Université Paris Cité and Université Sorbonne Paris Nord, Inserm, IAME, Paris, France; 4 Bonn Center for Mathematical Life Sciences & Life and Medical Sciences Institute, University of Bonn, Bonn, Germany; 5 Deutsches Zentrum Immuntherapie (DZI), Friedrich-Alexander-Universität Erlangen-Nürnberg and Universitätsklinikum Erlangen, Erlangen, Germany; 6 Bavarian Cancer Research Centre (BZKF), Friedrich-Alexander-Universität Erlangen-Nürnberg and Universitätsklinikum Erlangen, Erlangen, Germany; Pennsylvania State University, UNITED STATES OF AMERICA

## Abstract

Understanding the mechanisms that govern viral spread in human airway epithelium (HAE) remains a major challenge, particularly with regard to identifying and quantifying key factors such as cell type-specific infectivity, viral transmission paths, and the innate immune dynamics. Although mathematical models and experimental advances have provided valuable insights into respiratory infections, revealing the complex spatio-temporal interactions of infection and immune processes on a tissue-level have remained elusive. Here, we present a novel workflow that combines time-resolved bulk measurements and spatially-explicit image information to allow the inference of viral and immune kinetics within HAE for respiratory viruses. While standard inference methods typically require custom summary statistics and resourceful fitting procedures for each individual data set, our workflow relies on the combination of different, simulation-based trained neural networks using *BayesFlow*, a framework for neural posterior estimation that allows for amortized inference and the integrative analysis of multimodal data. We validated our approach by simulating viral infection dynamics in HAE using systems of increasing complexity that account for tissue heterogeneity, cell type-specific kinetics and interferon-mediated immune responses, mirroring experimental measurements. Thereby, we could show that integrating spatial information is essential to reliably infer viral transmission kinetics and innate immune interactions on a tissue-level. Applying our approach to experimental data on SARS-CoV-2 infection dynamics within HAE culture systems, we estimated that 84% [58%,100%] of all infections were due to cell-associated transmission, pointing towards local transmission as the dominant mode of SARS-CoV-2 spread within HAE. Our workflow can be readily applied to HAE culture systems for inference of viral and innate immune kinetics of different respiratory viruses, allowing multimodal data integration without the need for frequent resourceful re-fitting approaches.

## Author summary

Determining how respiratory viruses spread within human airway epithelium is an important pre-requisite to identify key processes that determine disease progression and potential therapeutic targets. Although experimental culture systems have increased our ability to study viral spread under physiological conditions, disentangling and quantifying the spatio-temporal dynamics of infection and immune processes still remains challenging. Here, we present a novel workflow that combines individual cell-based mathematical models with various spatial and time-resolved measurements. We validated the approach, which relies on simulation-based trained neural networks and allows the simultaneous integration and analysis of multimodal data, using synthetic data of different complexities. Applying it to SARS-CoV-2 infection dynamics within HAE culture systems indicated that local transmission dynamics is the dominant mode of SARS-CoV-2 spread within epithelium. The presented workflow provides a computationally efficient general framework to assess viral and immune dynamics within tissues for a diverse range of viral infections.

## Introduction

The spread of viral infections within tissues is the result of a complex interplay between tissue structure, viral intrinsic factors and counteracting immune responses. With viruses, such as influenza A or SARS-CoV-2, depending on the expression of particular cell-surface receptors to mediate viral entry [1,2], the distribution of different cell types within the targeted epithelium, as well as viral and cellular turnover dynamics, will all influence the establishment and progression of the infection. Localised effects and cell type-specific dynamics further play a role when it comes to viral transmission pathways [3,4], tissue pathology and regeneration [5], as well as innate immune dynamics, with for example the expression of interferon (IFN) observed to be cellular context dependent [6]. Therefore, understanding and disentangling the complex dynamics and kinetics of respiratory viral infections on a tissue and cellular level is essential in order to predict disease progression, and develop appropriate therapeutic strategies.

Advancements within experimental techniques, such as synthetic cultures or organoid systems, have increased our ability to study viral infections under physiological-like conditions [7,8]. These approaches provide us with various types of data, including time-resolved measurements of viral load and tissue pathology, as well as image based information from histology stainings or time-lapse microscopy, allowing us to study viral infections at an unprecedented level of detail. For example, using air-liquid-interface cultures of human airway epithelium and patient derived lung organoids increased our understanding on the cell type-specific infectivity and infection dynamics of SARS-CoV-2 within tissue [9–11]. However, revealing the complex, intermingled dynamics of viral spread, tissue regeneration and innate immunity requires an integrative approach that combines the various types of measurements in order to identify and quantify the key processes that drive viral infection and disease progression.

Mathematical modeling has been essential in inferring viral infection kinetics [12]. Combining mathematical models and experimental or clinical data has provided key insights into viral turnover dynamics and appropriate therapeutic strategies for various viruses [13,14]. While the majority of analyses has been based on bulk measurements, such as viral load and infected cell count, using systems of ordinary differential equations [12], the notion on the importance of tissue effects for infection progression has led to an increased development of mathematical modeling approaches that describe viral infection dynamics on a tissue level. Computational frameworks, such as CompuCell3D [15], Morpheus [16] or cellular automaton models [17–19], allow to model the dynamics of viral infections within multi-cellular environments. These frameworks that rely on cellular Potts models (CPM) [16,20] or other lattice-based approaches [18,21] enable to account for spatial heterogeneity and specific tissue structures when modeling infection dynamics. Besides using these tools to theoretically study spatio-temporal infection dynamics within respiratory tissues [18,19,21,22], there have now been increasing attempts to perform data-driven modeling by using appropriate parameter inference methods in order to identify and quantify tissue-related viral infection kinetics. For example, by combining time-lapse microscopy data and bulk measurements of HIV-1 spread within 3D *ex vivo* tissue cultures with a CPM of the dynamics, we could infer HIV-1 transmission dynamics on a single cell level and determine, how different environments influence these dynamics [23]. Similar approaches have been used to infer the transmission dynamics of hepatitis C virus (HCV) from cell culture systems [24], as well as the growth dynamics of tumor spheroids [25] or cell migration patterns [26].

Given the intrinsic stochasticity within these systems, as well as the difficulty to formulate an explicit likelihood-function for optimisation, approximate Bayesian computation (ABC) has been the method of choice in order to perform data-driven modeling and parameter inference [23–25,27]. The likelihood-free approach also enabled the integrative analysis of time-resolved bulk and time-limited image data during model adaptation, with each of the different types of data providing parts of the information necessary to understand and infer the spatio-temporal dynamics of infection within the particular tissue [23,24]. Spatial information provided by snapshots of the infection have been found to be essential in order to understand viral transmission dynamics on a tissue-level, with different metrics, such as cluster sizes and distributions of infected cells, used to characterise the infection pattern [24,28]. However, parameter inference by ABC methods still requires a pre-defined weighting on the contribution of these different measurements and summary statistics for model adaptation, which will rely on prior-assumptions and influence model performance, as well as the inferred kinetics. In addition, despite parallelisation strategies and efficient combination of modeling and inference frameworks (e.g., *FitMultiCell*) [25,27,29], these approaches experience substantial computational costs, particularly associated with the required iterative model simulations given the complexity of the multi-scale and multi-cellular dynamics considered.

In order to overcome these limitations, we here present a novel workflow that uses amortized Bayesian inference to infer viral and immune kinetics within human respiratory epithelium. It especially allows for multimodal data integration by simultaneously combining time-resolved bulk measurements and spatially explicit image information during the evaluation procedure. The approach relies on a framework for neural posterior estimation (i.e., *BayesFlow* [30]) that combines different neural networks, including a summary and a conditional invertible neural network, which are jointly trained using simulations. In addition, it allows for amortized inference, i.e., the application to various data sets without the necessity for computationally expensive re-fitting procedures.

Validating and benchmarking our approach against synthetic data considering various infection scenarios, we show that the approach is able to robustly infer and quantify viral kinetics on a tissue level. Application to experimental data of SARS-CoV-2 infection within HAE culture systems allowed us to reveal viral and innate immune kinetics and indicated that cell-associated transmission is the dominant mode of SARS-CoV-2 spread within human airway epithelium.

## Results

### An integrative workflow to infer viral and immune kinetics within human airway epithelium

**Fig 1:**
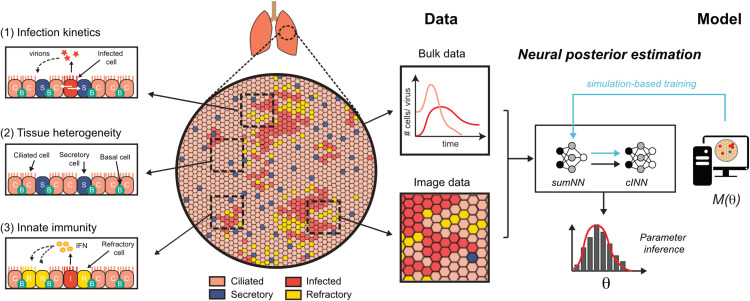
Sketch of the complex dynamics and the developed workflow to infer viral spread within HAE: With the complex dynamics of respiratory viral spread within HAE being influenced by (1) viral-specific transmission modes and infection kinetics, (2) tissue heterogeneity and regeneration dynamics, and (3) innate immune responses, an integrative approach is developed to infer and disentangle these dynamics by simulation-based Bayesian inference allowing the combination of multimodal data. Thereby, interference relies on two interconnected neural networks, i.e., including a summary (*sumNN*) and conditional invertible (*cINN*) neural network that are trained using simulated data.

To infer the complex, intermingled dynamics of viral and innate immune processes within human airway epithelium from standard experimental measurements, we developed a workflow that is able to simultaneously evaluate time-resolved bulk measurements and image data (Fig 1). By connecting time courses of viral load, IFN concentrations and tissue pathology, e.g., measured by the transepithelial electrical resistance (TEER), with spatially-resolved and individual cell-based measurements on infection patterns, we aim at retrieving the relevant information of local and global dynamics in order to assess the infection dynamics on a tissue level.

The approach relies on the concept of simulation-based Bayesian inference [31]. Similar to analysing viral dynamics by mathematical models, it requires the assumption of a mechanistic framework that provides a mathematical description of the assumed underlying processes [12]. To this end, we extended and adapted our previous multi-cellular and multi-scale cellular Potts model of HAE [22] to simulate the spatio-temporal dynamics of viral and immune dynamics on a tissue-level. From the simulations we can generate synthetic analogues of experimental measurements, including time-resolved standard population-level metrics for infection spread and tissue integrity (e.g., viral load, TEER), as well as image-based information that provide spatially-resolved measurements of infection patterns (e.g., abundance and distribution of clusters of infected cells) corresponding to histological images (S1 Text).

In contrast to likelihood-based (e.g., Maximum-Likelihood-Estimation) and traditional likelihood-free methods (e.g., Approximate Bayesian Computation [27]), parameter inference is based on the Bayesian neural parameter estimation framework *BayesFlow* [30]. The workflow consists of two interconnected neural networks, i.e., a summary and a conditional invertible neural network (cINN), that are jointly trained using simulated data [30,32]. By simulating the model *M* for various parameter combinations θ to generate synthetic data, a summary network is trained to learn characteristic summary features of these observed data, followed by the cINN to learn a bijective mapping between the parameter combinations θ and a latent distribution conditioned on the simulated observations. After the simulation-based training phase, the (inverted) cINN is then applied to the actual data, providing estimates of the Bayesian posterior distribution of θ given the observed data (Fig 1). The approach allows for multimodal data integration without the necessity to define or weight specific summary statistics to balance the consideration of local (spatial) and global (bulk) information (see also S1 Text, as well as [30] for further details on the method).

In the following, we validated the general applicability of our approach using models of increasing complexity to determine the ability of inferring kinetic parameters that describe infection spread by accounting for (1) viral-specific transmission modes and infection kinetics, (2) tissue heterogeneity and regeneration dynamics, and (3) innate immune responses (Fig 1). Thereby, we also examined how effectively kinetic parameters can be determined under different observational constraints across a broad range of infection scenarios.

### Integrating spatial information is essential to infer viral transmission kinetics within tissues

**Fig 2:**
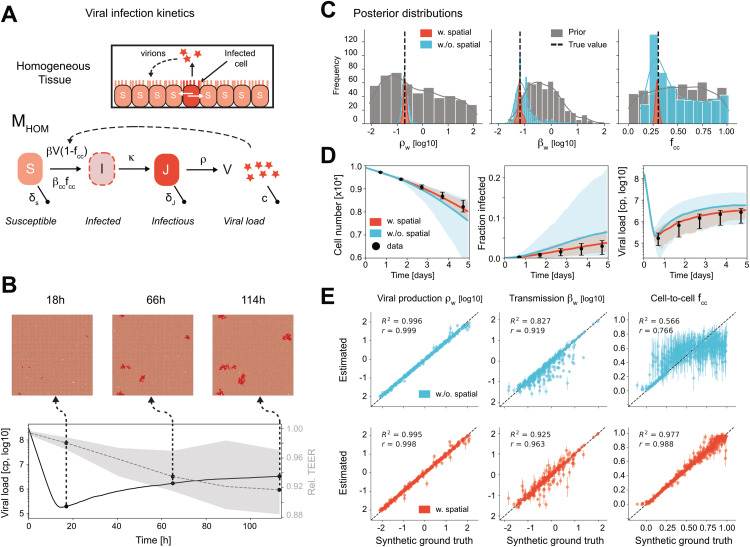
Inferring viral kinetics in homogeneous tissues: **(A)** Sketch of the processes of viral infection as considered in the *M*_*HOM*_-model distinguishing between susceptible, *S*, infected, *I*, and infectious cells, *J*, and assuming homogeneous tissue conditions without tissue regeneration. **(B)** Progression of the infection and snapshots of the spatial distribution of infected cells for one particular parameter combination θ across *n* = 10 simulations. Measurements are obtained at 18, 42, 64, 90 and 114 h post infection. **(C)** Prior (grey bars) and posterior distributions obtained by the developed framework indicating inference of ground-truth parameters (dashed line) that control viral production, $\rho_{w}$, transmission, $\beta_{w}$, or transmission modes, *f*_*cc*_, with (red bars) and without (blue) considering spatial image information in the inference procedure. The inference for one particular parameter combination $\theta =(\rho_{w},\beta_{w},f_{cc})$ is shown. **(D)** Simulated dynamics showing mean (solid line) and min-max prediction band across 100 simulations with the best parameter combination θ given the respective inference conditions. The synthetic data used for model analysis are shown in black (mean (dot) and standard deviation (whiskers)). **(E)** Global accuracy of parameter inference across 500 simulated ground-truth parameter sets for each of the three different parameters considered based on the Pearson correlation coefficient, *R*^2^.

In a first step, we evaluated the effectiveness of the simulation-based amortised Bayesian inference approach in determining viral infection kinetics within homogeneous tissues. To this end, we developed a spatially-explicit CPM implementation of a target-cell limited model, *M*_*HOM*_, which represents a simplified version of the previously developed model [22], and only distinguishes between susceptible (*S*), infected (*I*) and infectious cells (*J*) (Fig 2A). Susceptible cells can become infected by either (i) cell-free infection dependent on the extracellular viral load (*V*) and a transmission parameter $\beta_{cf}$, or (ii) the direct contact to infectious neighbouring cells regulated by a transmission parameter $\beta_{cc}$, with the relative contribution of these two modes further controlled by *f*_*cc*_. Upon infection, infected cells experience a viral eclipse phase determined by the transition parameter κ before they become infectious and start producing viral particles (ρ). In this scenario, tissue pathology is mediated by the death of susceptible and infectious cells, without considering tissue regeneration (see also *Materials and Methods* and [22] for further details).

The model *M*_*HOM*_ was then used to generate synthetic data that mimick experimental measurements, i.e., simulating the infection dynamics and obtaining bulk and image-based information, such as viral load, TEER and the distribution of infected cells at particular time points (Fig 2B). As we are particularly interested in the ability of inferring parameter describing viral kinetics, *M*_*HOM*_ was simulated given different combinations for the parameters governing viral production, infectivity and transmission, i.e., $\theta =(\beta_{w},\rho_{w},f_{cc})$, while all other parameters were kept fixed (see S1 and S2 Tables). Hereby, $\beta_{w}$ and $\rho_{w}$ define scaling factors for the viral transmission and production rates, respectively, in comparison to the standard parameterisations adopted from [22]. We generated 10^4^ training samples with each relying on a unique parameter combination θ, which was simulated several times to account for disrupted sampling (S1 Text). Using simulation-informed prior distributions for the individual parameters assists the choice of the sample size to balance computational costs and simulated cell numbers (S1 Text).

With variations in the relative contribution of viral transmission modes, i.e., *f*_*cc*_, showing strong effects on the size, abundance and spatial distribution of clusters of infected cells only visible within image data (S1 Fig), we specifically examined how the integration of spatially-resolved image information influences the ability of inferring viral infection kinetics. To this end, our Bayesian Inference approach was trained either with or without the spatial information on the distribution of infected cells provided by image-based analyses on the synthetic data. This included the average cluster size and abundance, the distance between individual clusters, as well as the average number of infected neighbours per cell, with details provided in S1 Text.

Training both networks on bulk and spatial information showed a remarkable ability in inferring the parameters describing viral spread within the tissue, given by a notable contraction in the posterior estimates compared to the prior distribution for each individual parameter (Fig 2C), which also includes parameters that were considered less frequent in the prior distributions used for training, i.e., considering rare infection patterns (S2 Fig). This could also be seen in the model capacity of re-simulating the ground-truth infection dynamics, with predictions being able to recover similar uncertainty as observed in the synthetic ground-truth data (Fig 2D). Global accuracy for all parameters given by the correlation between the ground-truth and inferred parameters across a validation set of 500 different parameter combinations not considered within the training phase reached *R*^2^ > 0.9 (Fig 2E, $\rho_{w}$ — *R*^2^ = 0.995; $\beta_{w}$— *R*^2^ = 0.925; *f*_*cc*_ — *R*^2^ = 0.977). In contrast, neglecting spatial information leads to larger uncertainty, especially for the parameter *f*_*cc*_ describing local infection dynamics (Fig 2C-2E), with a decrease in the global precision to almost half compared to a situation including spatial information ($f_{cc}\;-\;-R^{2}=0.566$). Thus, the presented approach is able to reliably infer viral infection kinetics within homogeneous tissues from experimental based measurements, with the integration of image-based information being essential to reliably infer local transmission kinetics.

### Tissue heterogeneity and regeneration dynamics do not impair inference of viral kinetics

**Fig 3:**
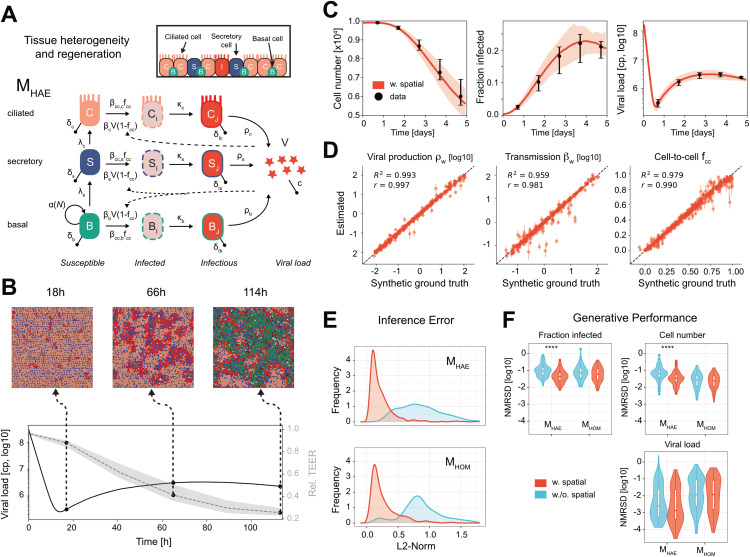
Inferring viral kinetics in heterogeneous tissues. **(A)** Processes and their characteristic parameters considered within the *M*_*HAE*_-model resembling HAE by distinguishing between basal, *B*, secretory, *S*, and ciliated cells, ***C*.** Tissue regeneration and cell-type specific infection kinetics are additionally included. **(B)** Simulated viral spread in HAE with snapshots obtained at different time points. **(C,D)** Prediction accuracy of inferred parameter estimates describing viral kinetics, $\theta =(\beta_{w},\rho_{w},f_{cc})$, using the *BayesFlow*-framework and including spatial measurements. Predictive checks for the cell and viral load dynamics for one particular parameter combination θ **(C)**, and global accuracy of parameter inference across 500 synthetic ground-truth values (**D**) are shown. **(E)** Distribution of inference error for the *M*_*HOM*_ and *M*_*HAE*_-model with and w./o. spatial information calculated by determining the L2-norm between inferred and synthetic ground-truth parameters across the 500 validation samples per condition. **(F)** Comparison of the generative performance of neural posterior estimators for *M*_*HOM*_ and *M*_*HAE*_ in resembling viral load and cell dynamics determined by the root mean squared difference across the simulated time series from 10 parameter combinations sampled from the posterior.

To further assess how cell heterogeneity and tissue regeneration influence the inference of viral infection kinetics, we repeated our analyses by using an extended model that accounts for the heterogeneity and structure of human airway epithelium, *M*_*HAE*_. The model represents our previous CPM of HAE [22] and distinguishes between basal (*B*), secretory (*S*), and ciliated (*C*) cells that are organised within a pseudo-stratified epithelium. Cells are characterised by individual cell type-specific infection and viral production kinetics, with the model additionally accounting for tissue regeneration and cell differentiation pathways that follow proliferating basal to secretory and subsequently ciliated cells (Fig 3A, see also *Materials and Methods*, and [22] for a full description of the model and its implementation). In contrast to the original version [22], the impact of innate immunity is neglected at this point.

Consistent with our previous analysis, *M*_*HOM*_, we were interested in the ability to infer viral infection kinetics in these more complex conditions. In this case, the unknown infection kinetics were defined by $\theta =(\beta_{w},\rho_{w},f_{cc})$, with $\beta_{w}$ and $\rho_{w}$ describing weighting factors that scale the cell type-specific viral transmission, $\beta_{x}$, and production parameters, $\rho_{x}$, x∈{B,S,C}, by maintaining their relative proportion. The individual processes of tissue regeneration and cell type-specific infection were parameterised using previous estimates obtained from experimental data of HAE cultures [22,33] (S2 Table). As before, the model system was used to generate synthetic data resembling experimental observations, with tissue heterogeneity leading to more variable spatial patterns of infection compared to *M*_*HOM*_ due to the cell type-specific infection dynamics (Fig 3B).

Applying our Bayesian inference approach trained on a large set of synthetic data to various test cases indicates a similar robustness in capturing the underlying dynamics and variation (Fig 3C), and global precision in parameter inference (Fig 3D) as observed for *M*_*HOM*_ across a large range of infection scenarios. Including spatial information in the analytical procedure was thereby essential to infer global and local infection dynamics, substantially decreasing the inference error (Fig 3E), which is determined by the L2-norm between inferred and given ground-truth parameters across the 500 synthetic validation samples, and the prediction error of inferred parameter combinations (Fig 3F) (S1 Text). Thereby, even infrequent and limited spatial information of just two time points is sufficient to allow a robust assessment of tissue kinetics, with intermediate and later time points seeming to be more informative when robust patterns have already emerged (S3 Fig). Thus, the efficacy of the *BayesFlow* inference approach in inferring the underlying viral transmission kinetics remains unaffected by the added complexity due to tissue heterogeneity and regeneration dynamics if these latter processes can be pre-determined.

### Simultaneous inference of viral and innate immune kinetics requires additional prior knowledge

**Fig 4:**
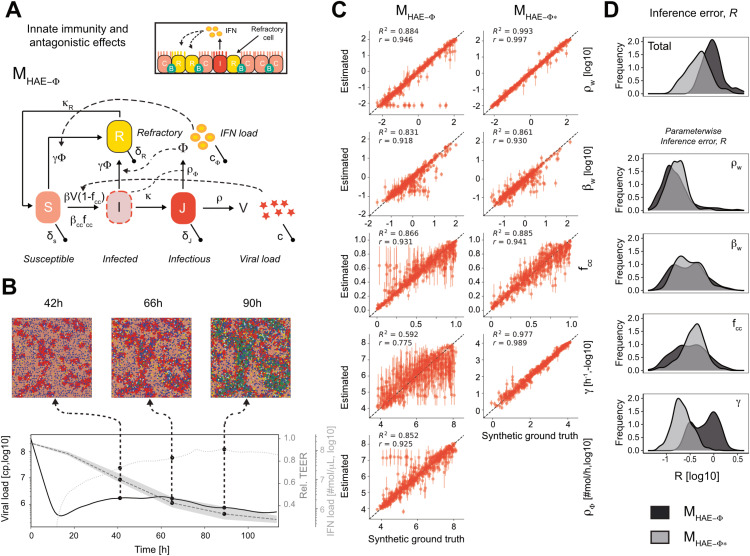
Simultaneous inference of viral and innate immune kinetics: **(A)** Sketch of the additional processes describing innate immune dynamics by IFN-expression within the $M_{HAE-\Phi }$-model accounting for autocrine and paracrine effective modes. **(B)** IFN load and fraction of cells refractory to infection for one simulated parameter combination $\theta =(\rho_{w},\beta_{w},f_{CC},\rho_{\Phi },\gamma )$. **(C)** Global accuracy of parameter inference based on the correlation of estimated and ground-truth parameters across 500 individual simulated parameter combinations θ using the Bayesian inference approach. Accuracy is shown for a scenario with an unknown ($M_{HAE-\Phi }$, left column) and known value ($M_{HAE-\Phi ⋆}$, right column) for the IFN–production rate, $\rho_{\Phi }$, with increased performance visible across all parameters in the latter scenario. **(D)** Combined and parameter-wise inference error showing the decreased uncertainty in parameter estimates for the innate immune kinetics in $M_{HAE-\Phi ⋆}$ compared to $M_{HAE-\Phi }$.

With the previous approaches neglecting innate immunity, we further assessed the ability to infer viral and innate immune kinetics from standard experimental measurements given the intermingled dynamics of these processes within tissues. To this end, the *M*_*HAE*_-model was extended to account for the local auto- and paracrine effective dynamics of type-1 IFN signaling. IFN is assumed to be actively produced by infected and infectious cells with a cell type-specific parameter $\rho_{\Phi }$, with the local IFN concentration, Φ, constantly degraded by $c_{\Phi }$. IFN acts by (i) inhibiting infected cells from becoming infectious and (ii) rendering susceptible cells refractory to infection, with both processes described by the effectiveness parameter γ. The transient protective state has an average duration of $1/\kappa_{\Phi }$ (see Fig 4A and *Materials and Methods*). This extended model $M_{HAE-\Phi }$ accounts for antagonistic interactions of viral and immune dynamics within HAE culture systems that affect infection patterns, and also provides bulk measurements of the IFN-concentration (Fig 4B).

Evaluating the ability of the Bayesian inference approach to simultaneously infer viral and immune dynamics — described by the unknown parameters $\rho_{w},\beta_{w}$ and *f*_*CC*_ for viral infection kinetics as before, and $\rho_{\Phi }$ and γ for IFN production and effectiveness — we found that parameter inference is impaired even given spatial information on the infection pattern (Fig 4C). Based on the additional complexity and feedback dynamics, global accuracy of individual parameter inference decreased ranging from $R^{2}\sim 0.6-0.88$ across all parameters. Especially the identification of parameters describing innate immune kinetics was impaired (Fig 4C). However, parameter inference for immune effectiveness could be restored if prior knowledge on IFN-production can be obtained. Pre-defining the value of $\rho_{\Phi }$, model $M_{HAE-\Phi ⋆}$ leads to decreased uncertainty in parameter estimates (Fig 4D). Thus, additional bulk measurements of the total IFN concentration are not sufficient to simultaneously infer cell-based viral and immune dynamics without prior knowledge on specific aspects of the innate immune kinetics.

### Analysis of SARS-CoV-2 infection dynamics within HAE indicates dominant cell-associated transmission modes

**Fig 5:**
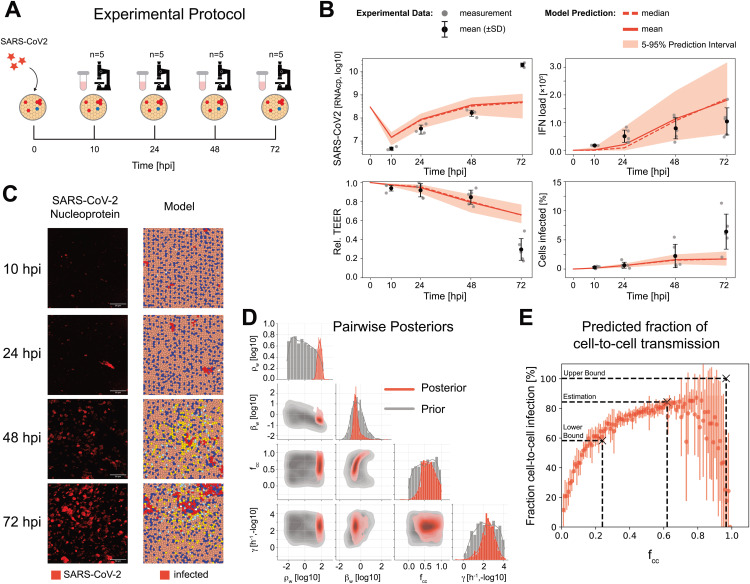
Application to experimental data: **(A)** Experimental protocol for analysing SARS-CoV-2 infection dynamics within HAE culture systems obtaining bulk and image measurements from HAE cultures of a single donor at 10, 24, 48 and 72 hpi, with 5 replicates per time point. **(B)** Experimental measurements (grey dots, mean±SD - black dots) and model predictions adapting model $M_{HAE-\Phi ⋆}$ to the experimental data (red lines/ shaded area) for different characteristic quantities (see also S4C Fig) and *Materials and Methods*). **(C)** Snapshots of the infection patterns at indicated time points of the experimental data and representative model simulations. **(D)** Parameter estimates showing posterior contraction (red) from the prior (grey) for the four parameters estimated. **(E)** Reference curve relating *f*_*cc*_ to the actual fraction of infected cells by cell-to-cell transmission based on model predictions. Our estimates for *f*_*cc*_ for SARS-CoV-2 predict 84% [58%,100%] of the infections being due to local transmission dynamics.

Having validated the general ability of the proposed approach in inferring viral infection kinetics on a tissue-level, we applied the method to experimental data on the infection dynamics of SARS-CoV-2 within human airway epithelium. Reconstituted epithelia of human primary airway epithelial cells obtained from one single donor were infected with SARS-CoV-2 and followed up to 72h post infection. At 0, 24, 48 and 72 hpi bulk and image measurements on viral load, tissue integrity and interferon concentration, as well as spatial patterns of viral infected cells were obtained (Fig 5A). Data indicated a continuous increase of viral load and the percentage of infected cells over the observed time period, reaching a considerable loss in tissue integrity at 72 hpi.

Adapting and re-training our model $M_{HAE-\Phi ⋆}$ to the experimental protocol (see *Materials and Methods*, and S4A and S4B Fig), model predictions show good agreement with the observed dynamics for the various quantities considered, except for underestimating the infection load and tissue destruction at 72 hpi, which is characterized by a sharp increase between 48–72 hpi (Fig 5B). This also applied to the characteristics of the spatial infection patterns observed (Figs 5C and S4C). The analysis showed considerable parameter contraction for the estimated parameters affecting viral production, transmission and IFN efficacy (Fig 5D), with estimates and their 95% credibility intervals provided in S3 Table.

With our model able to retrieve the particular transmission mode by which a cell got infected, we determined a reference curve relating *f*_*cc*_ to the actual fraction of cells infected by cell-to-cell transmission. Our estimated parameter range for *f*_*cc*_ indicated that 84% [58%,100%] of infections were due to cell-to-cell transmission, pointing towards local transmission dynamics as the dominant mode of SARS-CoV-2 spread within heterogeneous human airway epithelium (Fig 5E).

## Discussion

The assessment of infection and immune dynamics of respiratory viruses within human airway epithelium is essential to understand disease progression and decide on appropriate anti-viral strategies. Here, we validate and apply a novel inference approach for quantifying tissue-related viral and immune kinetics within respiratory epithelium by combining standard bulk and image-based measurements. With each of the various data types (e.g., viral load, TEER or histology) only assessing parts of the global and/or local infection dynamics, connecting the information within a mechanistic framework allows us to infer and quantify tissue-related dynamics at single cell resolution. While there has been a large body of literature on computational modeling frameworks that study the spatio-temporal dynamics of respiratory viruses considering various processes across multiple scales (e.g., [34,35]), approaches that combine these frameworks with experimental or clinical data are limited. This is partly due to the challenges for parameter inference tools associated with the complex and stochastic modeling frameworks that require substantial computational resources [27,29]. Here, we used a novel approach based on simulation-trained neural networks that allows the simultaneous combination of bulk and image-based measurements to assess viral and immune kinetics on a single-cell level.

Validating and benchmarking our approach against synthetic data using different levels of complexity, we could show that the ability to robustly infer and disentangle tissue-related viral transmission dynamics relies on the availability of image information. Although this information only originates from static snapshots of the infection, the obtained distribution of infection patterns is sufficient to assess transmission dynamics at tissue level, as it has also been shown before [28,36]. With image acquisition being experimentally demanding and costly, we could additionally show that appropriate accuracy can be achieved with images even obtained from just one or two time points. In extension to the previous approaches, we also validated that this inference holds true in more complex scenarios, including pseudo-stratified epithelium, cell heterogeneity and tissue regeneration dynamics, as well as antagonistic effects of innate immunity. However, in the latter scenario, the ability to simultaneously infer viral and immune kinetics is impaired, with the additionally measured bulk IFN-concentration being not sufficient to disentangle these dynamics. Prior-knowledge on parts of innate immune kinetics is required to restore inference ability. With image-based information already shown to be essential for inferring infection dynamics in homogeneous tissue conditions, similar information for single cell immune activity, as, e.g., obtained by fluorescent microscopy or spatial transcriptomics [4,6,37–40] could provide helpful information to simultaneously infer viral and innate immune kinetics within tissues.

With the simulation-trained neural network approach used here, the incorporated training of a summary network allows for an automated weighting of the summary statistics that combine bulk and spatial measurements, avoiding potential biases for certain key features when weights are pre-defined, as, e.g., required for standard ABC-approaches [23,24,28,29]. However, the approach still relies on the definition of manually determined quantities in order to describe infection patterns, such as the distribution and size of infected cell clusters that have already been found useful for inferring viral transmission pathways [24,28]. While *BayesFlow* would allow the use of summary networks that directly work on the image data itself, as, e.g., used in pattern recognition [30,41], simulation-trained summary networks would not be generalizable towards the real world data [42]. Alignment of the respective summary spaces by choosing appropriate spatial statistics as done here mitigates this problem [42]. Advancements to ensure the comparability of simulated and experimental image data, as, e.g., based on style adaptation and content preservation methods [43], could help to improve an unbiased integration of image-based information in the future.

Applying our approach to data of HAE culture systems infected by SARS-CoV-2, parameter estimates indicated that cell-associated transmission modes dominate SARS-CoV-2 spread within human airway epithelium, with our estimates of 84% [58%, 100%] being in agreement with previous estimates of 90% of infections being due to cell-to-cell transmission [44]. However, our estimates, which were obtained from HAE-samples from a single donor, still contain a large uncertainty with regard to the specific values of this dominance, with variations in donor tissue composition and individual host factors potentially affecting their generalisability. In addition, while we could show that the considered framework is able to reproduce the observed early infection kinetics across the multimodal measurements considered, our model especially underestimates the viral burden and disease pathology at 72 hpi. Testing several model assumptions that could affect this underestimation, including the consideration of viral release by burst upon cell death or with a time-dependent increase instead of a continuous production by infectious cells, as well as the explicit modeling of intracellular viral RNA and infectious virus to account for potential non-linear relationships [45–47], were not sufficient to explain this deviation. A potential explanation for the underestimation of viral burden and epithelial damage at later time points could be related to the modeling and calculation of tissue regeneration and integrity. Our previous analyses already indicated that regeneration dynamics might differ during infection and inflammation from the kinetics assumed for homeostatic conditions used here [22]. In addition, the calculation of the tissue integrity from the image data itself relying on obtained cell connectivities [22] might not sufficiently account for a potential non-gradual loss in TEER measurements due to holes or major breaks within the tissue. More detailed experimental analyses on tissue regeneration and cell differentiation dynamics within HAE culture systems would be necessary to reliably parameterise these processes and potentially support the connection of tissue integrity and infection dynamics.

Besides allowing multimodal data integration within a dynamic framework, the strength of the proposed parameter inference approach especially lies within the possibility for amortised inference. Having trained our framework only once given the underlying assumed modeling and data structure, we would be able to quickly apply it to several data sets spanning a large range of possible infection dynamics, without the necessity for repeated resourceful fitting procedures as required for standard inference approaches [27]. With the simulations of the complex spatio-temporal dynamics being computationally demanding to account for sufficient cell numbers, various conditions and disrupted sampling, using a general framework for network training amortizes applicability to several different scenarios. This also includes the robust consideration of different sampling regimes by providing normalised time vectors to train the summary network, with only the relative distance between time points being relevant [48]. While in our case the consideration of 10^4^ unique parameter combinations per condition used for training seems to be rather small compared to other studies relying on 10^5^ − 10^6^ training samples [42], the use of informed prior distributions supports the sufficiency of this sample size while maintaining computational efficiency.

In addition, simulation-based Bayesian inference further improves the evaluation procedure and amortisation by allowing the combination of experimental design and parameter inference, as well as model selection [49] and identification of patient-specific and population effects [50]. With the modeling framework enabling Bayesian experimental design (BED) by evaluating the information content of different experimental protocols [51,52], application to actually measured data directly benefits from the already performed training procedures. Thus, using a standard framework for viral dynamics within human airway epithelial tissue, our approach presented here could be readily applied to data from HAE culture systems to infer and quantify viral and innate immune kinetics for various respiratory viruses, including IAV, SARS-CoV-2 and RSV. However, specific model assumptions with regard to tissue regeneration dynamics and cell type-specific viral and innate immune kinetics might require further adaptation.

In summary, using the concept of neural posterior estimation, the approach presented here provides a framework for the experimental design and analysis of viral infections within different epithelia by combining bulk and spatial measurements. Using simulation-based trained neural networks, it allows the assessment of the sufficiency of obtained experimental data for inferring different kinetic processes. Although the particular model and obtained inference for respiratory infection kinetics within HAE introduced here still contains some limitations, the performed analyses show the general suitability and appropriateness of the considered workflow that combines individual-cell based models with standard experimental measurements to infer tissue-related infection dynamics. With the specific model and its underlying assumptions being easily exchangeable and adaptable to different kinds of tissues and infection scenarios, it presents a general framework for assessing viral and immune kinetics on a tissue-level.

## Materials and methods

### Mathematical models

We used three different mathematical models of increasing complexity to determine and validate the ability of inferring viral and immune kinetics by our workflow. All models relied on the cellular Potts modeling (CPM) framework developed in [22] to simulate viral infection dynamics in HAE-culture systems using the *Morpheus* software [16]. In the following, the general processes and parameters considered within each of the different models are introduced, with the specific implementations of the stochastic dynamics within the CPM explained in detail below.

### The target cell limited model for homogeneous tissue - *M*_*HOM*_

As a first approach, we considered a simplified target cell limited model, *M*_*HOM*_, to describe viral spread within a homogeneous tissue. The model distinguishes between susceptible, *S*, infected, *I*, and infectious cells, *J*. Susceptible cells can become infected either by diffusing viral particles dependent on the viral concentration, *V*, and a transmission parameter $\beta_{cf}$, or by cell-to-cell transmission via direct contact to infectious cells dependent on a transmission parameter $\beta_{cc}$, respectively. The probability to get infected by either of the two transmission modes is controlled by *f*_*cc*_, which controls for the relative contribution of cell-free and cell-to-cell transmission to the infection dynamics (see below). After an eclipse phase with an average duration of 1/κ, infected cells will become infectious and start producing viral particles by a viral production rate ρ. The loss of susceptible and infectious cells is characterised by the parameters $\delta_{S}$ and $\delta_{J}$, respectively, with the viral concentration *V* being reduced by the clearance parameter, *c*. For simplicity, proliferation of susceptible cells, as well as the loss of infected cells is neglected within this model. A sketch of the model is shown in Fig 1A. The following system of ordinary differential equations provides a mean-field approximation of the considered dynamics:


$$\begin{array}{c} \begin{array}{cc} \frac{dS}{dt}= & \;\;-\beta_{cf}V(1-f_{cc})S-\beta_{cc}f_{cc}JS-\delta_{S}S\\ \frac{dI}{dt}= & \;\;\beta_{cf}V(1-f_{cc})S+\beta_{cc}f_{cc}JS-\kappa I\\ \frac{dJ}{dt}= & \;\;\kappa I-\delta_{J}J\\ \frac{dV}{dt}= & \;\;\rho J-cV \end{array} \end{array}$$ (1)

However, please note that the actual dynamics follows a spatially explicit and stochastic implementation as outlined below.

### Modeling heterogeneous tissue dynamics - *M*_*HAE*_

As an extension to the simplified *M*_*HOM*_-model and to account for tissue heterogeneity, we used the human airway epithelium model, *M*_*HAE*_, as it has been developed by [22]. For a detailed description of the model please see [22]. In brief, the model distinguishes between basal, *B*, secretory, *S*, and ciliated cells, *C*, considering their individual turnover and epithelial regeneration. Basal cells continuously differentiate into secretory cells which further differentiate into ciliated cells. Each cell type is characterised by cell type-specific infection dynamics, regulating viral transmission (β), duration of the eclipse phase (κ) and viral production (ρ) with the mean-field approximation for each cell type described analogous to Eqs. (1) (see also Fig 2A). In contrast to the model presented within [22], refractory cell types were not included within the *M*_*HAE*_-model, as innate immune protection was neglected at this point.

### Incorporating innate immune dynamics - $M_{HAE-\Phi }$

To fully mimick the situation of viral spread within HAE-culture systems, we extended the *M*_*HAE*_-model to account for innate immune dynamics mediated by interferon (IFN). Upon infection, cells are assumed to start actively producing IFN after a lag-phase determined by an average duration $1/\kappa_{\Phi }$ with a cell type-specific parameter $\rho_{\Phi }$. The IFN concentration, Φ, rendering susceptible cells to become refractory to infection, and inhibiting infected cells from becoming infectious. Dependent on the ratio between κ and $\kappa_{\Phi }$, infected and infectious cells will be actively producing IFN. The protective cell state is assumed to be transient, with an average duration defined by $1/\kappa_{R}$, while the local IFN-concentration is degraded according to the parameter $c_{\Phi }$. A sketch showing the major processes of the IFN-dynamics considered within the $M_{HAE-\Phi }$ model for one particular cell type is shown in Fig 3A, with the corresponding mean-field approximation described by extending Eqs. (1) to


$$\begin{array}{c} \begin{array}{c} \frac{dS}{dt}=-\beta_{cf}V(1-f_{cc})S-\beta_{cc}f_{cc}JS-(\delta_{S}+\gamma_{\Phi })S+\kappa_{R}R\\ \frac{dI}{dt}=\beta_{cf}V(1-f_{cc})S+\beta_{cc}f_{cc}JS-(\kappa +\gamma_{\Phi })I\\ \frac{dJ}{dt}=\kappa I-\delta_{J}J\\ \frac{dV}{dt}=\rho J-cV\\ \frac{dR}{dt}=\gamma_{\Phi }(I+S)-(\kappa_{R}+\delta_{R})R\\ \frac{d\Phi }{dt}=\rho_{\Phi }J-c_{\Phi }\Phi \end{array} \end{array}$$ (2)

For simplicity of notation, Eq. 2 considers $\kappa >\kappa_{\Phi }$, i.e., only infectious cells are producing IFN. This assumption is neglected in the stochastic, individual cell-based implementation below. Details with regard to the implementation of the different processes are explained below.

### Cellular Potts model implementation in Morpheus

The different models were implemented within the cellular Potts modeling (CPM) framework *Morpheus*, a multi-scale and multi-cellular modeling environment allowing to simulate the spatio-temporal dynamics of individual cells [16]. The CPM is a lattice-based model with each cell comprising several grid sites that dynamically change their shape by aiming to reach and/or maintain a predefined target volume and surface area controlled by the parameters volume strength, *V*_*S*_, and aspherity, Ψ. The basic model for human airway epithelium as presented by [22] (https://morpheus.gitlab.io/model/m6296/) was adapted to the different scenarios of the *M*_*HOM*_, *M*_*HAE*_ and $M_{HAE-\Phi }$-model. In brief, the model considers a monolayer of cells (*M*_*HOM*_) or pseudo-stratified epithelium ($M_{HAE},M_{HAE-\Phi }$), with the latter achieved by allowing basal cells to be “overgrown” by secretory and ciliated cells due to a lower volume strength *V*_*S*_ for the former cell type.

To simulate the stochastic dynamics of the various processes within the different models we assumed a constant hazard model, with the specific parameters, as, e.g., for cell proliferation, differentiation and death, used to calculate the probability of an event within a time step of the simulation. For example, the probability of a cell to die within a discrete time step Δt is calculated by $p_{\delta }(\Delta t)=1-e^{-\delta \Delta t}$. For cell death, cell differentiation and the duration of the viral eclipse phase, we additionally accounted for *k* = 4 intermediate compartments to simulate Γ-distributed dwell- and lifetimes. Please refer to [22] for more details on the implementation. In the following, we will particularly explain the implementation of viral infection and immune dynamics, with the consideration of innate immunity deviating from the original implementation in [22].

### Viral infection

Viral infection was implemented as previously described in [22] distinguishing between the two primary transmission mechanisms, i.e., cell-free and cell-to-cell infection. For cell-free infection, viral particles are produced at each grid site of infectious cells, *J*, at a constant rate ρ. These particles can spread throughout the system, modelled by a diffusive field of extracellular viral particles, with the effective viral diffusion rate set to $D_{V}=60\;\mu m^{2}/h$ [22,24]. The probability of a cell *i* to get infected within a time period Δt is then determined by $p_{cf}(\Delta t)=1-e^{-\beta V_{i}\Delta t}$, with *V*_*i*_ denoting the total concentration of viral particles across the cell body.

For cell-to-cell transmission, the probability of a cell to get infected, *p*_*cc*_, is determined by the number of adjacent infectious cells, *N*_*j*_, and calculated by $\begin{array}{c} p_{cc}=\beta_{cc}\sum {\mathrm{\backslash nolimits}}_{x}\rho_{cc,x}N_{j,x} \end{array}$. Hereby, the relative infectivity parameter $\rho_{cc}$ controls the transmission efficiency of the infecting cell (i.e., comparable to the viral production), and $\beta_{cc}$ the infectiveness of the receiving cell. The index *x* denotes the different cell types (i.e., basal, secretory, ciliated) when accounting for tissue heterogeneity.

In addition, a scaling factor *f*_*cc*_ is considered, which adjusts the relative contribution of each mode to viral spread. This leads to the overall transmission parameters $\tilde{\beta }=(1-f_{cc})w_{cf}\beta$ and ${\tilde{\beta }}_{cc}=f_{cc}w_{cc}\beta_{cc}$, with *w*_*cf*_ and *w*_*cc*_ denoting two weighting factors to adapt inferred bulk infection kinetics to the spatially-explicit modeling framework (see [22]). Please note that the values for β, $\beta_{cc}$, ρ and $\rho_{cc}$ are considered cell type-specific and parameterised according to [22] for *M*_*HAE*_ and $M_{HAE-\Phi }$, while using the parameterisations for ciliated cells in *M*_*HOM*_ (see also S2 Table).

In order to test various viral kinetics, we additionally considered two scaling factors, $\beta_{w}$ and $\rho_{w}$ that scaled the cell type-specific rates $\tilde{\beta }$ and ρ, respectively, while maintaining the relative ratios of cell type-specific viral production and transmission. Thus, the unknown parameters θ addressing viral kinetics in the different models are defined by $\theta =(\beta_{w},\rho_{w},f_{cc})$.

### Innate immunity

To consider innate immune response dynamics, the *M*_*HAE*_-model was extended by considering the production and secretion of IFN by infected and infectious cells. Upon infection, infected cells experience a lag-phase and randomly progress towards an IFN-productive state with probability $p_{\Phi }(\Delta t)=1-e^{-\kappa_{\Phi }\Delta t}$, considering *k* = 4 intermediate compartments that lead to Γ-distributed lag-times. During this time, infected cells can also turn infectious, which in theory allows infected and infectious cells to be IFN-productive dependent on the ratio of κ and $\kappa_{\Phi }$. Actively IFN-producing cells will produce IFN with a constant production rate $\rho_{\Phi }$, increasing the local extracellular IFN concentration, Φ, at all grid sites covered by the cell. The IFN-concentration, Φ, is modelled as an extracellular field of IFN particles which diffuse trough the simulated epithelium at a diffusion rate, $D_{\Phi }$, which was set to $D_{\Phi }=6\times 10^{2}\;\mu m^{2}/h$, based on previous analyses indicating a 10-times higher diffusion than for virions [37,53].

To account for the anti-viral and protective effects, i.e., autocrine and paracrine modes of IFN-signaling, the local IFN-concentration Φ, which we use as a surrogate for IFN-signaling strength, influences (i) the viral production rate of infectious cells, (ii) the probability of infected cells to turn infectious, and (ii) the susceptibility of uninfected cells to infection. For each infectious cell *J*_*i*_, the viral production rate ρ is reduced dependent on the local IFN-concentration across the cell, $\Phi_{i}$, according to a Hill-function given by


$$\begin{array}{c} \tilde{\rho }=\rho \frac{1}{1+{\left(\frac{\Phi_{i}}{IC_{50}}\right)}^{\nu }}. \end{array}$$ (3)

Hereby, *IC*_50_ denotes the IFN-concentration required for inhibiting half of the maximal viral production rate ρ, and ν the Hill coefficient. To account for the paracrine mode of IFN-signaling, susceptible and infected, but not yet infectious cells can turn into a refractory state, *R*, which prevents them from getting infected or becoming infectious, respectively. The probability of turning refractory also depends on the local IFN-concentration, $\Phi_{i}$, and is calculated by $p(\Delta t)=1-e^{-\gamma \Phi_{i}\Delta t}$ with γ defining a transition parameter. The refractory state is assumed to be transient with average duration $1/\kappa_{R}$, and refractory cells become susceptible again with probability $p(\Delta t)=1-e^{-\kappa_{R}\Delta t}$. Please note that for loosing the refractory status, we also use the concept of compartmentalisation, i.e., assuming *k* = 4 intermediate steps before making the transition to allow for Γ-distributed transition times (see above). To account for the antagonistic effect of the viral load on the IFN-signaling capacity, the degradation rate of IFN is linearly dependent on the logarithm of the viral load at the corresponding grid sites with $\begin{array}{c} {\tilde{c}}_{\Phi }=\ln(V)c_{\Phi } \end{array}$. In addition, the kinetics of interferon production or transition into refractory states is assumed to be not cell type-specific.

### Default model parameterization

In contrast to the CPM modelling framework shown in [22], which mimicked a radial cell-culture system, each of the different models (*M*_*HOM*_, *M*_*HAE*_ and $M_{HAE-\Phi }$) was implemented in a rectangular grid with periodic boundary conditions. This was done for simplicity without loss of generality and transferability to actual experimental data. Each system considers a total number of 10^4^ cells in an area of ~1.163 *mm*^2^ given an average cell density of $\sim 4.7\times 10^{4}$ cells, similar to the cell density used in [22]. In order to account for the changed structure of the model, the initial viral load, *V*_0_, was adapted, starting each simulation with $V_{0}\approx 1.8\times 10^{3}$ viral copies per $\begin{array}{c} \mu m^{2} \end{array}$. As before, simulations were performed with a time-step size of Δt=0.25h (=15 min) and simulating the dynamics for 500 time steps, i.e., 125 hours in real time. Infection was initialised after a burn-in phase of 6h to let the tissue reach a steady-state. All parameters relating to cell type-specific cell sizes, i.e., volume strength and aspherity, cell turnover and differentiation, as well as viral infection kinetics were referred from [22], i.e., being largely SARS-CoV-2 specific (S1 and S2 Tables). For the model *M*_*HOM*_, a monolayer of ciliated cells was assumed.

Inference of viral kinetics and innate immune dynamics focused on the parameters $\theta =(\beta_{w},\rho_{w},f_{cc},\rho_{\Phi },\gamma )$ dependent on the particular model considered. The full list of parameters describing the general structure of the CPM and the model-specific parameters are given in the Supporting Information (S1 and S2 Tables, respectively). All simulations were performed within Morpheus version v2.3.3 with the source code of the model available at *https://morpheus.gitlab.io/model/m6296/* and *https://github.com/GrawLab/SARS-ALIculture*.

### Parameter inference by BayesFlow

For parameter inference of immune and infection kinetics combining time-resolved spatial and bulk measurements, we used the Bayesian neural parameter estimation framework *BayesFlow* [30]. The approach relies on Bayesian posterior estimation by a combination of different neural networks and uses model-based training to allow inference of the posterior distribution of an unknown set of parameters θ from a given data set. *BayesFlow* represents a computationally efficient method, as, e.g., in comparison to approximate Bayesian computation [27], and refrains from the necessity of defining appropriate summary statistics. Here, we briefly introduce the approach with detailed explanations of various steps given within the Supporting Information (S1 Text). For full details on the theoretical aspects and the implementation of *BayesFlow*, we refer the reader to the original publication [30].

The *BayesFlow* architecture consists of two modules comprising two jointly trained neural networks: (i) a sequence network module which combines a filtering network based on a 1D-convolutional layer and a long-short term memory (LSTM) recurrent network which provide a summary network that condenses high-dimensional observational data into a fixed-length summary vector, i.e., summarising characteristic spatial and bulk information dependent on the given kinetics; and (ii) an inference module which relies on a conditional invertible neural network (cINN) to learn a bijective mapping between parameter sets and a latent space conditioned on the observational data.

*BayesFlow* relies on simulation-based training. During the training phase, both networks are jointly trained by model simulations for a large variety of parameter combinations θ, creating similar output as expected from possible experimental measurements (see S1 Text). Thereby, the invertible neural network maps latent variables from a normal distribution to the parameters $\theta_{i}$ conditioned on the simulated observations *X*_*i*_. This enables the network to learn the joint distribution $\left(X_{i},\theta_{i})\sim p(X|\theta )p(\theta \right)$. In the subsequent inference phase, the trained network is used to approximate the posterior distribution over parameters given real observational data *x*_obs_, that is, $q(\theta |X=x_{\text{obs}})\approx p(\theta |X=x_{\text{obs}})$. Owing to the amortized nature of *BayesFlow*, this inference step is fast and does not require re-training, provided that the data have the same structure as those used during training. Unlike traditional likelihood-free methods, such as approximate Bayesian computation (e.g., pyABC) [27], *BayesFlow* enables rapid inference after the initial training phase. This makes it especially well-suited for applications involving repeated inference tasks, such as estimating kinetic and immunological parameters from diverse datasets addressing viral infection dynamics.

The generation of training data, the specifications of the *BayesFlow* network architecture, as well as the methods for assessing and qualifying parameter inference as used within this study are outlined in detail in S1 Text. The prior distributions and calibration curves, as well as hyperparameters for the *BayesFlow* architectures for each of the different models considered are shown in S5-S8 Figs and S4 Table.

### Experimental data

#### Reconstituted human airway epithelial model.

MucilAir HAE, reconstituted from human primary cells obtained from bronchial biopsies, was provided by Epithelix and maintained at an air-liquid interface in a specific culture medium in Costar Transwell inserts (Corning), according to the manufacturer’s instructions. The bronchial HAE used in this study was derived from a single donor, a 64-year-old Caucasian female non-smoker (batch number MD0786). HAE were infected with a Wuhan-like SARS-CoV-2 strain (BetaCoV/France/IDF0571/2020) [10] at a multiplicity of infection (MOI) of 0.01. At five time points (0, 10-, 24-, 48-, and 72-hours post-infection [hpi]), the impact of viral infection on epithelial integrity was assessed by measuring transepithelial electrical resistance using a dedicated volt-ohm meter (EVOM2, WPI) and expressed in ohms/cm^2^. At each time point, the basal medium was collected and analyzed by ELISA to measure IL-6 (Ref. 3460-1H-6, Mabtech) or IFN-λ secretion (Ref. 88-7296-22, Invitrogen). Viral genome quantification was performed by one-step real-time quantitative reverse transcription polymerase chain reaction (RT-qPCR) from total RNA extracted with the RNeasy Mini Kit (QIAGEN), as previously described [10]. The final time point of 72 hpi was chosen as a late stage resembling advanced infection, as interpretability of viral replication becomes impaired at later time points due to significant mortality already visible within the increased tissue destruction.

#### Confocal microscopy.

After fixation in 4% PFA, samples were permeabilized with PBS containing 0.1% Triton X-100 and blocked in the same buffer supplemented with 1% FBS. A primary antibody (mouse monoclonal anti-SARS-CoV-2 nucleoprotein N, Ref. MA529981, Invitrogen) was applied for 2 hours at room temperature, followed by washes and incubation with a fluorescent secondary antibody (goat anti-mouse Alexa Fluor 633, Ref. A21052, Invitrogen). Nuclear (4’,6-diamidino-2-phenylindole, DAPI, Ref. 66248, Invitrogen) and membrane (wheat germ agglutinin, WGA, Alexa Fluor 488, Ref. W11261, ThermoFisher Scientific) counterstains were then applied. Finally, membranes were excised from the inserts, mounted in Fluoromount-G (Ref. 00-4958-02, ThermoFisher Scientific), and imaged using a confocal microscope (Leica TCS-SP5X, CIQLE, Université Claude Bernard Lyon 1).

#### Image analysis.

Experimental confocal microscopy images were analysed by performing a 3D segmentation of nuclei based on the DAPI-channel using Cellpose (v.3.0.8, [54]) with a pre-trained cyto-model and an estimated diameter of 35 pixels. For all cell nuclei masks, the center of mass (CoM) was determined with scikit image (v.0.25.2), and sorted according to their z-position. To distinguish apical from basal cells, cells were classified using an adaptive filtering approach, after 3D images were divided into 400 tiles along the x- and y-axes. For each tile, the z-positions of the CoMs were scanned, starting at the most apical position and classifying all cells above/below the current cell as apical/basal until 80% of the cells in a tile were classified as apical. The threshold was selected based on prior analyses of HAE tissue cultures stained with the membrane protein ZO1.

To detect and quantify clusters of infected cells, Voronoi tesselation was performed for apical cells based on their CoMs by adapting the scipy-Voronoi tesselation algorithm (v.1.11.1) in order to recreate tissue connectivity. In addition, the viral nucleoprotein intensity within the NP channel was analysed, using a 2D maximum intensity projection followed by Gaussian filtering with a 7-pixel radius and Yen’s global thresholding to generate binarized infection maps. Cells were classified as infected if intersections of Voronoi tiles and binarized infection regions showed at least 50% overlap for the particular cell, with this threshold lowered to 25% if the detected total fraction of infected cells was less than 1%. The fraction of infected cells was calculated as the ratio of infected Voronoi tiles to all cell nuclei. Spatial level metrics of infected cell clusters, including cluster abundance, size and distances, as well as their variations and the number of infected neighbors, were obtained as based on the simulated data (S1 Text). To this end, the filtered Voronoi tiles were segmented and dilated.

This image analysis workflow was performed in Python (v.3.11.5) using the packages as described above, with image segmentation performed on an A100 40 GB NVIDIA GPU.

### Parameter inference for experimental data

The parameter inference workflow was adapted to the experimental data, using the framework and prior information of the $M_{HAE-\Phi *}$-model for generating training data. Snapshots of the simulation were captured at 10, 24, 48, and 72 hpi, using random crops representing approximately 2500 cells to mimic the number of cell nuclei observed on average in the microscopy data. To account for a possible selection bias during image acquisition that would favour selecting regions of infected cells, random crops with at least one infected cell cluster were considered in the training data. The derived spatial-level metrics were combined with measurements of viral load, IFN load, and TEER. For comparison with simulation data, the IFN load was converted using a factor of 2.751 × 10^4^ particles/pg mL^−1^, assuming a 2.5% efficiency in antiviral induction. TEER values were normalized to the highest measurement.

The new inference networks were trained on *n* = 10^4^ synthetic datasets, followed by hyperparameter tuning with the architectures for both, the summary and cINN, adjusted to enhance the inference performance (see Supporting Information S1 Text). To assess the inference accuracy of these networks, simulated posterior retrodictive checks were performed in a two-step process. In a first step, 10 parameter combinations were sampled from the estimated posterior distribution to generate disrupted synthetic datasets. Model fits were evaluated by calculating the root mean squared distance (RMSD) between simulated and experimental data. The top 10 network architectures were selected and considered in a second retrodictive check that involved the sampling of 100 parameter combinations from each estimated posterior, with 20 simulations performed for each selected combination. Mimicking the experimental data, individual data sets were generated by randomly sampling from different parameter combinations and replica, resulting in five random replicates per time point and measurement. The process was repeated 100 times to generate 100 distinct datasets for each network architecture. The best fitting network architecture was selected by calculating the mean RMSD between simulations and experiments across the 100 disrupted datasets, and evaluating the network bias using the AUC-ECDF metric. To estimate cell-to-cell infection events during SARS-CoV2 infection in the experimental data, a calibration curve was generated to determine the relationship between the parameter *f*_*CC*_ and cell-to-cell infections. This involved sensitivity analysis of the *f*_*cc*_ parameter, keeping the other parameters constant across 100 simulations with 10 replicates each, followed by fitting a power law function. The estimated *f*_*CC*_ posterior distribution values were then used to calculate the fraction of cell-to-cell infection events given the calculated power law.

## Supporting information

S1 Text Parameter inference by BayesFlow. (PDF)

S1 Fig Spatial infection patterns: Time courses/Examples of *M*_*HOM*_ considering different values for the cell-to-cell transmission scaling factor *f*_*cc*_. All other parameters are given as in S2 Table.
(PDF)

S2 Fig Reliable inference of rare infection dynamics. (**A**-**D**) Examples of posterior distributions for each of the different models considered that indicate the ability to robustly infer viral and innate immune kinetics, even if these were only rarely considered within the prior-distributions during training.
(PDF)

S3 FigInfluence of experimental design on retrieving tissue-specific infection dynamics.(**A**) Sketch of experimental design assuming different scenarios in the time coverage and number of replicates at which image information can be obtained. (**B**) Inference error *R* determined by the L2-norm of the estimated posterior means θ from the ground truth given 100 validation sets for the different image acquisition scenarios considered that vary in the number of time points (*n* = 5) or replicates (*t* = 5) using model *M*_*HAE*_. (**C**) Average relative performance of parameter inference determined by the relative inference error compared to a scenario having full image coverage and sampling depth (all, *t* = 5, *n* = 5) for various scenarios of data acquisition.(PDF)

S4 FigBayesFlow-calibration and adaptation of $M_{HAE-\Phi ⋆}$ to SARS-CoV-2 in HAE.(**A**) Empirical cumulative distribution functions (ECDF) and (**B**) recovery plot for 500 validation sets of estimated parameters for the model $M_{HAE-\Phi ⋆}$ trained and adapted to the experimental protocol used for analysing the spread of SARS-CoV-2 within the HAE culture systems. (**C**) Experimental measurements (grey dots, mean±SD - black dots) and model predictions adapting model $M_{HAE-\Phi ⋆}$ to the experimental data (red lines/ shaded area) for additional characteristic spatial quantities that were not shown in Fig 5B.(PDF)

S5 Fig BayesFlow-calibration *M*_*HOM*_. (**A**) Empirical cumulative distribution functions (ECDF) and (**B**) prior distribution for the estimated parameters of model *M*_*HOM*_.
(PDF)

S6 Fig BayesFlow-calibration *M*_*HAE*_. (**A**) Empirical cumulative distribution functions (ECDF) and (**B**) prior distribution for the estimated parameters of model *M*_*HAE*_.
(PDF)

S7 FigBayesFlow-calibration $M_{HAE-\Phi }$.(**A**) Empirical cumulative distribution functions (ECDF) and (**B**) prior distribution for the estimated parameters of model $M_{HAE-\Phi }$.(PDF)

S8 FigBayesFlow-calibration $M_{HAE-\Phi }⋆$.(**A**) Empirical cumulative distribution functions (ECDF) and (**B**) prior distribution for the estimated parameters of model $M_{HAE-\Phi ⋆}$.(PDF)

S1 Table CPM parameters. General parameters for the cellular Potts model (CPM) describing human airway epithelium.
(PDF)

S2 Table Dynamic parameters. Parameters used within the different model systems to describe viral, immune and tissue regeneration dynamics.
(PDF)

S3 TablePosterior estimates for SARS-CoV-2 spread in HAE culture systems.Posterior estimates for the parameters describing viral and immune dynamics for the experimental SARS-CoV-2 HAE-culture systems using model $M_{HAE-\Phi ⋆}$ adapted to the experimental protocol.(PDF)

S4 Table Optimal hyper-parameters for neural network architecture and training. Optimal hyper-parameters identified for each of the different models and training data considered.
(PDF)

S1 Data Experimental data of SARS-CoV-2 infection: Experimental data of HAE cultures of a single donor infected with SARS-CoV-2. The data contains the different bulk and spatial measurements as used for analysis.
(XLSX)
