# Supplementary material for: Multimodal data integration to determine viral and innate immune kinetics in human airway epithelium
Source: PLoS Comput Biol. 2026 May 20;22(5):e1014248. doi: 10.1371/journal.pcbi.1014248 (PMC13245872; doi:10.1371/journal.pcbi.1014248)
Supplement: S1 Text — (PDF) [file pcbi.1014248.s001.pdf]

S1 Text: Parameter inference by BayesFlow

The BayesFlow approach

To test the ability of inferring the parameters describing immune and infection kinetics from experimental-like data combining time-resolved spatial and bulk measurements, we used the Bayesian neural parameter estimation framework *BayesFlow* [1]. In the following, we will explain in detail the generation of the training data for the various models, the hyperparameter tuning, as well as the methods used for assessing and qualifying parameter inference. For full details on the theoretical aspects and the implementation of *BayesFlow*, we refer the reader to the original publication [1].

Creating training data

For the simulation-based training of the *BayesFlow* neural networks, we sampled 10^4 different parameter combinations θ for each of the different models considered. This implied the parameters $\boldsymbol{\theta} = (\beta_w, \rho_w, f_{cc})$ addressing viral kinetics for the models M_{HOM} and M_{HAE}, and the additional consideration of the innate immune dynamics for model $M_{HAE-\Phi}$, i.e., $\boldsymbol{\theta} = (\beta_w, \rho_w, f_{cc}, \gamma, \rho_\Phi)$. We choose a number of 10^4 samples for training in order to balance computational costs and simulated cell numbers, given the amount of simulations necessary for each of the various conditions by accounting for disrupted sampling and image acquisition. The choice of the sample size was supported by sampling parameter combinations from pre-defined prior ranges (see below and S5-S8 Fig.). Mimicking experimental scenarios, simulations were evaluated at 5 time points representing 18, 42, 66, 90 and 114 h after infection generating spatial and population-based measurements.

Bulk and spatial measurements: We assessed three population-level metrics, including the viral load and the TEER, as well as the total IFN-concentration (for $M_{HAE-\Phi}$). While viral load and IFN-level were immediately taken from the outputs generated by *Morpheus*, the TEER was determined based on the generated images and using the method explained in detail in [2]. In addition, to characterise the spatial patterns, four spatial-level metrics were determined: (i) the average size of clusters of infected cells and their standard deviation, (ii) the cluster abundance, (iii) the average distance between clusters plus standard deviation, and (iv) the average number of infected neighbours per cell and the standard deviation. Each of these metrics was assessed by using an image processing pipeline that analysed the snapshots from the *Morpheus* simulations obtained at the indicated time points. To this end, infected regions were segmented using the chosen cell color coding to identify clusters of infected cells, with the number of separated areas, as well as the number of infected cells per cluster, providing the information for determining cluster abundance and average cluster size. Distances between clusters were determined by calculating the center of mass for each of the identified infected regions and computing the corresponding distance matrix. Finally, the number of infected neighbouring cells of each cell was obtained directly from *Morpheus* using the contact logger [3]. Image analysis was performed using the `pyclesperanto`-package 0.24.4 and `scikit-image` 0.23.2 within python 3.10.24.

Disrupted sampling: With experiments usually requiring disrupted sampling, in which independent tissue cultures are analysed at consecutive timepoints to obtain image and bulk measurements, our training data were generated accordingly. A total of eight replicates were run for each parameter combination $\boldsymbol{\theta}$ with each replicate analysed at the indicated time points to obtain the specific data structure as described above. For each time point, five simulations were randomly sampled to construct the final training data set for each parameter combination $\boldsymbol{\theta}$. This approach allowed us to resemble disrupted sampling by maintaining computational efficiency.

Generating simulation-informed prior distributions

With overall infection dynamics being sensitive to the choice of the parameters defining viral and immune kinetics, i.e. leading to instant infection of the whole culture or no spread of infection at all, simulations were used to pre-determine prior-ranges that lead to realistic and informative infection dynamics. To determine appropriate prior-distributions for M_{HOM}, we performed a grid-search for the parameters describing the infection kinetics, $\theta = (\beta, \rho, f_{cc})$, comprising a total of 10^4 unique parameter combinations θ_i with $\beta \in [10^4, 10^5]$, $\rho \in [10^4, 10^5]$ and

$f_{cc} \in [10^4, 10^5]$. For each parameter combination θ_i a simulation was performed and the maximum fraction of infected cells, f_{max}, over the whole simulated time period, as well as the time point at which f_{max} was reached, t_{max}, was determined. A parameter combination was considered to lead to realistic infection dynamics if the peak of infection was reached more than $24h$ after start of infection with less than 90% of cells getting infected, i.e., $f_{max} < 0.9$ and $t_{max} > 24h$. A Gaussian kernel density estimator was applied to the filtered parameter combinations θ_i to determine simulation-based prior distributions for β, ρ and f_{cc}.

The same approach was adapted to generate simulation-informed prior distributions for the M_{HAE}-model, as well as to determine prior-ranges for the parameters describing the immune kinetics within the $M_{HAE-\Phi}$-model. For the latter, an additional grid-search was performed for the two parameters γ and ρ_F, generating 10^3 unique parameter combinations with $\gamma_i \in [10^4, 10^5]$ h^{-1} and $\rho_{\Phi,i} \in [10^4, 10^5]$ mol/h. These parameters were combined with random samples from the pre-determined prior-distributions of the infection parameters, (β, ρ, f_{cc}) obtained for M_{HAE}, leading to 10^4 unique parameter combinations $\theta_i = (\beta_i, \rho_i, f_{cc,i}, \gamma_i, \rho_{\Phi,i})$. As before, simulation-informed prior distributions for the $M_{HAE-\Phi}$-model were then obtained by subsequent simulation, filtering and application of Gaussian-Kernel density estimation.

BayesFlow network architecture and training

For each of the different models, *BayesFlow* was trained on the synthetic data generated with a total of 10^4 unique parameter combinations that were sampled from the simulation-informed prior distributions (see above). Training was performed in BayesFlow-Version 1 using the standard offline fitting routine with an ADAM optimizer with the default cosine decay for the learning rate and a batch size of 256. The required training data were pre-simulated and stored on disk to avoid on-the-fly data generation. During training, we performed simultaneous hyperparameter tuning for the parameters defining the architecture of the summary and conditional invertible neural network (cINN) within *BayesFlow* to optimize evaluation performance. This involved the following hyperparameters and variations: (a) the length, l, of the one-dimensional summary vector for the summary network with $l = 6, 8$ or 10; (b) the dropout rate p considering $p = 0, 0.05$ or 1; and (c) the number of coupling layers of the cINN with $n = 4, 6$. Considering a learning of rate of $\eta = 0.0005$, this leads to 18 different network architectures, i.e. hyperparameter combinations ψ_j, $j = 1, \dots, 18$, that were evaluated for each model M_x. In addition, we used 100 epochs and saved all models in between to determine the best model.

To determine if our trained neural posterior estimators are well-calibrated, we employed simulation-based calibration (SBC) based on 500 validation parameter sets θ for each model M_x [4]. Calculation of the deviation of the empirical cumulative distribution function (ECDF) from a perfectly uniform CDF, we can detect any miscalibration or deviation present in our trained neural estimator [4, 5]. Consequently, the accuracy of the posterior estimator was evaluated by the area under the curve of the derived ECDF curve (AUC-ECDF) (see S5-S8 Fig). To select the best-performing *BayesFlow* network architecture for each model M_x, we determined for each hyperparameter combination ψ_j the number of epochs, E, that led to the least miscalibration error according to the sum of the AUC-ECDF. Subsequently, we ranked the performance across the resulting hyperparameter combinations $\psi_{E,j}$ by additionally considering the global inference accuracy, Λ. The calculation of Λ was based on calculating the Pearson correlation coefficients $R^2(\theta_i)$ for each of the individual model parameters in $\theta = (\theta_1, \dots, \theta_n)$ based on the 500 selected validation sets using the mean of the posterior distributions provided for θ_i, which was obtained by calculating the mean over 500 randomly sampled point estimates (see also [1]). The global accuracy Λ is then given by

$$\Lambda = n - \sum_{i=1}^{n} R^2(\theta_i) \tag{1}$$

with n defining the number of unknown parameters that are estimated for each of the different models considered. For $\Lambda = 0$, we would have perfect inference of the ground truth. The definition of the global accuracy Λ was chosen to weight each parameter equally.

The performance of each hyperparameter combination $\psi_{E,j}$ is then defined by the two measures of AUC-ECDF and Λ. All $\psi_{E,j}$, $j = 1, \dots, 18$ were independently ranked for both measures, with the best performing network architecture then determined by selecting $\psi_{E,j}$ with the minimal rank sum across the individual ranks. S4 Table shows the hyperparameter selected for each of the different models.

Adaptation to experimental data

For the application to the actual experimental measurements of SARS-CoV-2 viral spread within HAE culture systems, the workflow described above for hyperparameter testing was slightly adapted. This involved adjusting the length of the summary vector l with $l = 10, 14, 18, 22, 26, 30$, the dropout rates p considered to $p = 0.1, 0.2, 0.3$, the number of cINN coupling layers with $n = 4, 6, 8, 10$, and the learning rate with $\eta = 0.0001, 0.0005$, resulting in 143 different network architectures.

Evaluating parameter inference and goodness of fit

Inference error: To evaluate the accuracy of inferring parameters of viral and innate immune kinetics given different model assumptions and available data, we determined the inference error R by calculating the L2-norm between the estimated parameter combinations $\hat{\theta}$, given by the mean of the posterior distributions provided by *BayesFlow*, against the ground truth value θ, i.e., $R = \left\|\hat{\theta} - \theta\right\|_2$. By calculating R across 500 validation sets, we obtain a distribution for the inference error for each of the different models and scenarios considered (compare Fig 3E, Fig 4D). In addition, we also determined a parameter-wise inference error given by $R(\theta_i) = \sqrt{(\hat{\theta}_i - \theta_i)^2}$.

Prediction error: To assess the generative performance of the neural posterior estimator, i.e., determining how well the inferred parameter combinations could reproduce the originally observed dynamics, we randomly sampled 10 parameter combinations out of the obtained posterior distributions and re-simulated the dynamics given the respective model M_x with model performance evaluated by the normalised root-mean-squared error across the simulated time series.

Average relative inference error: In addition, to compare the influence of various experimental designs differing in time coverage and sampling frequency with regard to image acquisition on the inference accuracy, we calculated the average relative inference error $\overline{RR}$ (S3 Fig). To this end, the inference error of the particular protocol, $R_{\text{exp},i}$ was compared to the inference accuracy of the protocol assuming full coverage, $R_{\text{all},i}$, across all $i = 1, \ldots, n$ validation sets considered according to

$$\overline{RR} = \frac{1}{n} \sum_{i=n}^{100} \frac{R_{\text{exp},i}}{R_{\text{all},i}}$$

$\overline{RR}$ calculates the relative average increase in the inference error for the adapted protocol. For testing various experimental designs, we calculated the average relative inference error across $n = 100$ validation sets for each condition.

References

1. Radev ST, Mertens UK, Voss A, Ardizzone L, Kothe U. BayesFlow: Learning Complex Stochastic Models With Invertible Neural Networks. IEEE Trans Neural Netw Learn Syst. 2022;33(4):1452–1466. doi:10.1109/TNNLS.2020.3042395.

2. Raach B, Bundgaard N, Haase MJ, Starruß J, Sotillo R, Stanifer ML, et al. Influence of cell type specific infectivity and tissue composition on SARS-CoV-2 infection dynamics within human airway epithelium. PLoS Comput Biol. 2023;19(8):e1011356. doi:10.1371/journal.pcbi.1011356.

3. Starruß J, de Back W, Brusch L, Deutsch A. Morpheus: a user-friendly modeling environment for multiscale and multicellular systems biology. Bioinformatics. 2014;30(9):1331–1332. doi:10.1093/bioinformatics/btt772.

4. Talts S, Betancourt M, Simpson D, Vehtari A, Gelman A. Validating Bayesian Inference Algorithms with Simulation-Based Calibration; 2020. Available from: `http://arxiv.org/abs/1804.06788`.

5. Modrák M, Moon AH, Kim S, Bürkner P, Huurre N, Faltejsková K, et al. Simulation-Based Calibration Checking for Bayesian Computation: The Choice of Test Quantities Shapes Sensitivity. Bayesian Analysis. 2025;20(2). doi:10.1214/23-BA1404.
