# Supplementary figures and images for: Multimodal data integration to determine viral and innate immune kinetics in human airway epithelium
Source: PLoS Comput Biol. 2026 May 20;22(5):e1014248. doi: 10.1371/journal.pcbi.1014248 (PMC13245872; doi:10.1371/journal.pcbi.1014248)

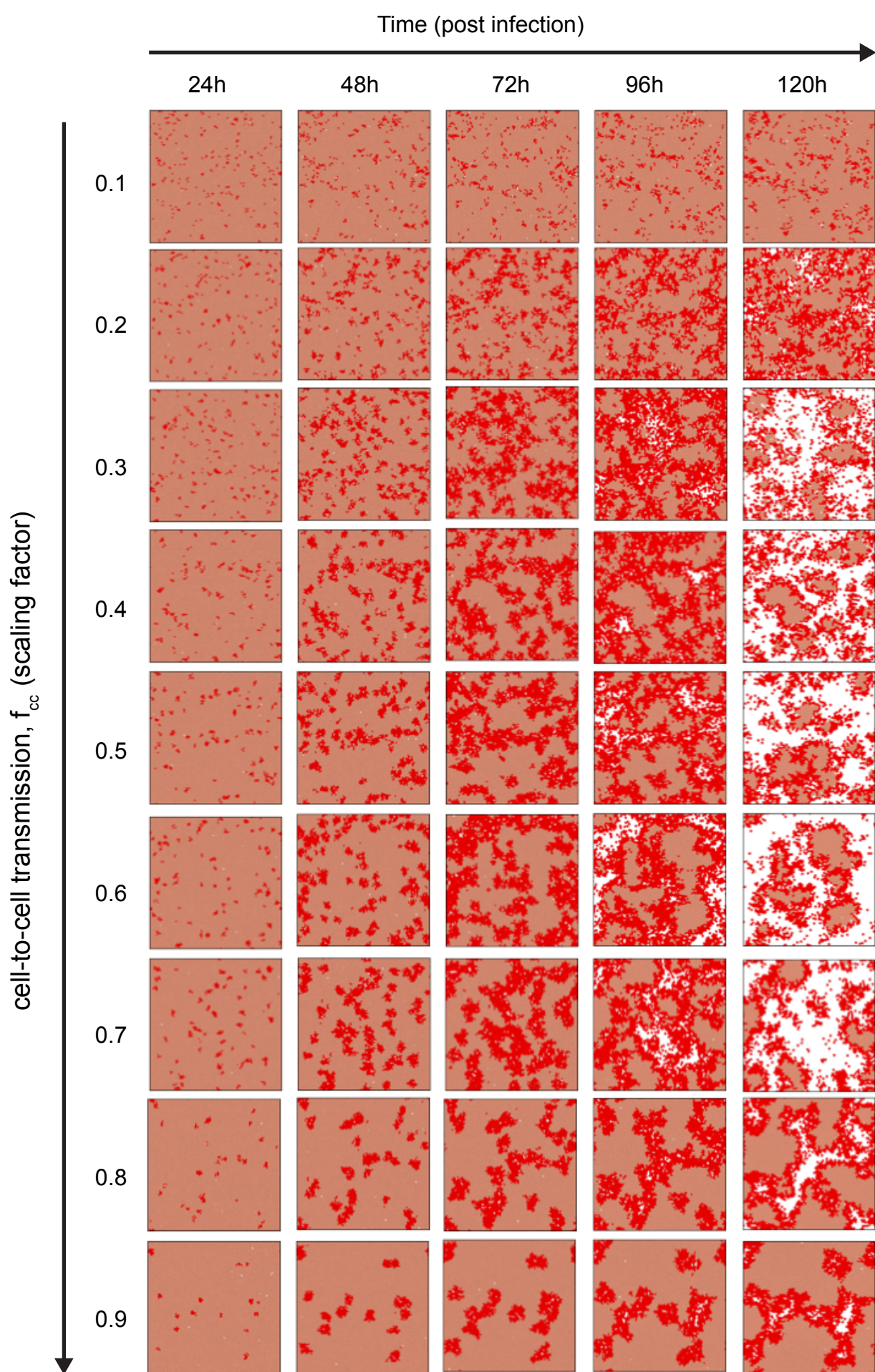

Supplement: S1 Fig — All other parameters are given as in S2 Table. (PDF) [file pcbi.1014248.s002.pdf]

**A** $M_{\text{HOM}}$ 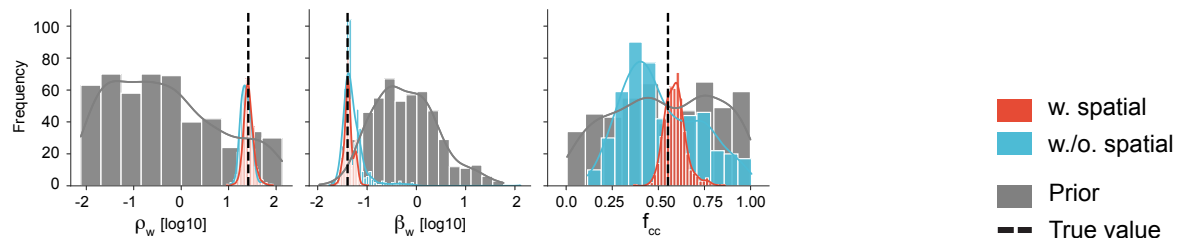**B** $M_{\text{HAE}}$ 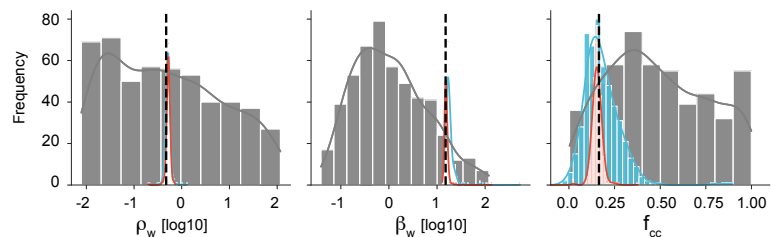**C** $M_{\text{HAE}-\Phi}$ 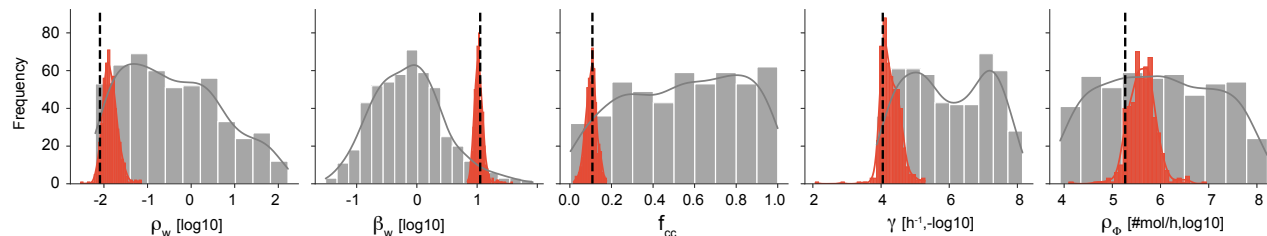**D** $M_{\text{HAE}-\Phi^*}$ 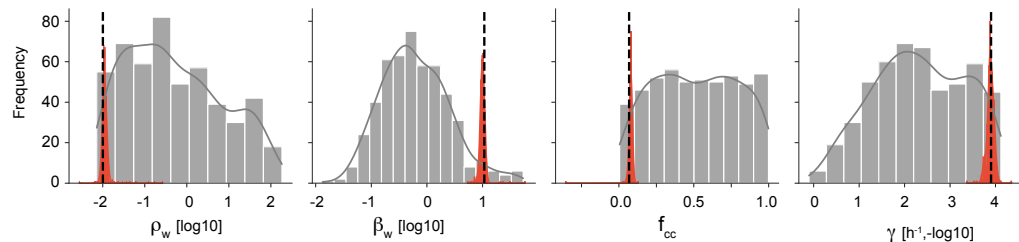

Supplement: S2 Fig — (A-D) Examples of posterior distributions for each of the different models considered that indicate the ability to robustly infer viral and innate immune kinetics, even if these were only rarely considered within the prior-distributions during training. (PDF) [file pcbi.1014248.s003.pdf]

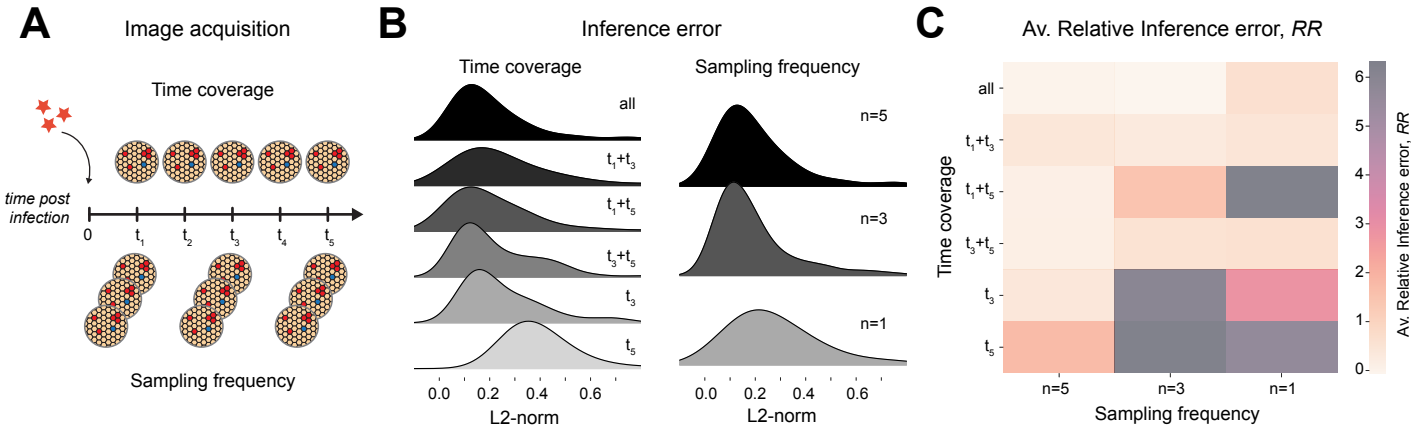

Supplement: S3 Fig — (A) Sketch of experimental design assuming different scenarios in the time coverage and number of replicates at which image information can be obtained. (B) Inference error R determined by the L2-norm of the estimated posterior means θ from the ground truth given 100 validation sets for the different image acquisition scenarios considered that vary in the number of time points (n = 5) or replicates (t = 5) using model MHAE. (C) Average relative performance of parameter inference determined by the relative inference error compared to a scenario having full image coverage and sampling depth (all, t = 5, n = 5) for various scenarios of data acquisition. (PDF) [file pcbi.1014248.s004.pdf]

**A**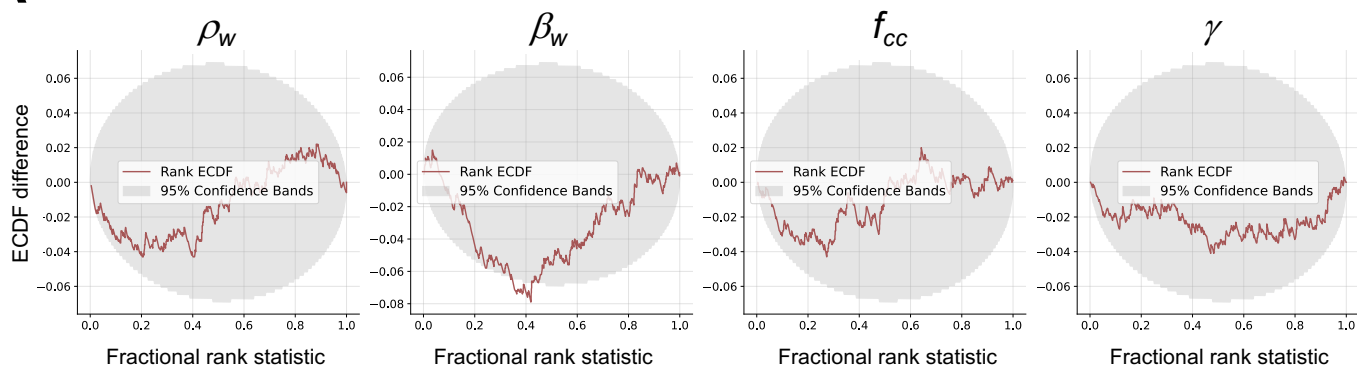**B**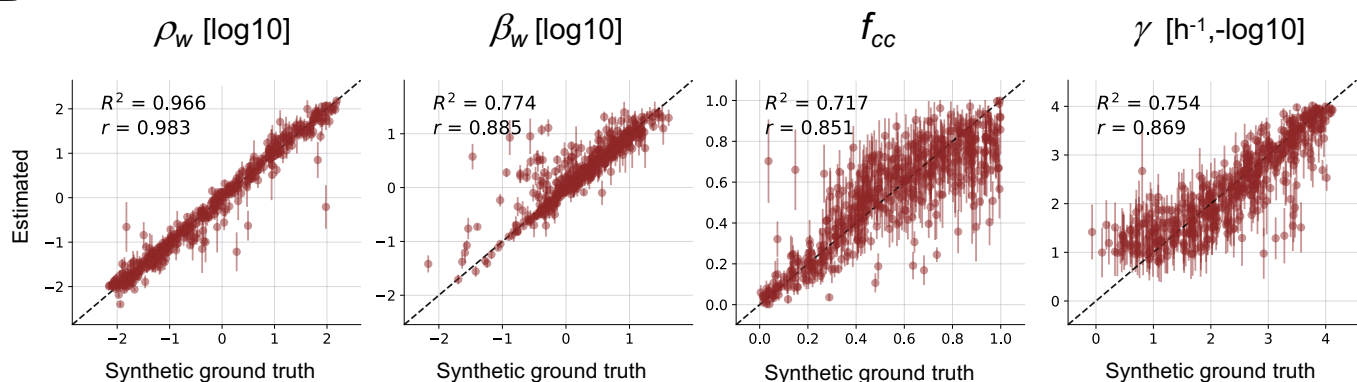**C**

### Spatial metrics (Experimental data & model predictions)

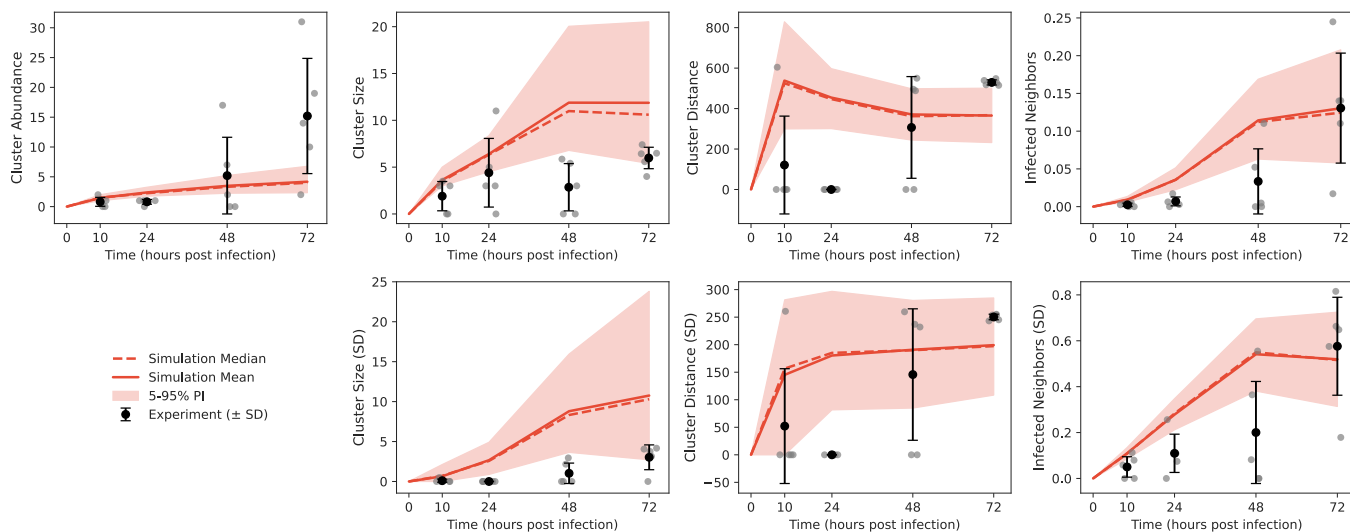

Supplement: S4 Fig — (A) Empirical cumulative distribution functions (ECDF) and (B) recovery plot for 500 validation sets of estimated parameters for the model MHAE−Φ⋆ trained and adapted to the experimental protocol used for analysing the spread of SARS-CoV-2 within the HAE culture systems. (C) Experimental measurements (grey dots, mean±SD - black dots) and model predictions adapting model MHAE−Φ⋆ to the experimental data (red lines/ shaded area) for additional characteristic spatial quantities that were not shown in Fig 5B. (PDF) [file pcbi.1014248.s005.pdf]

**A** $M_{\text{HOM}}$ 

w/o. spatial

with spatial

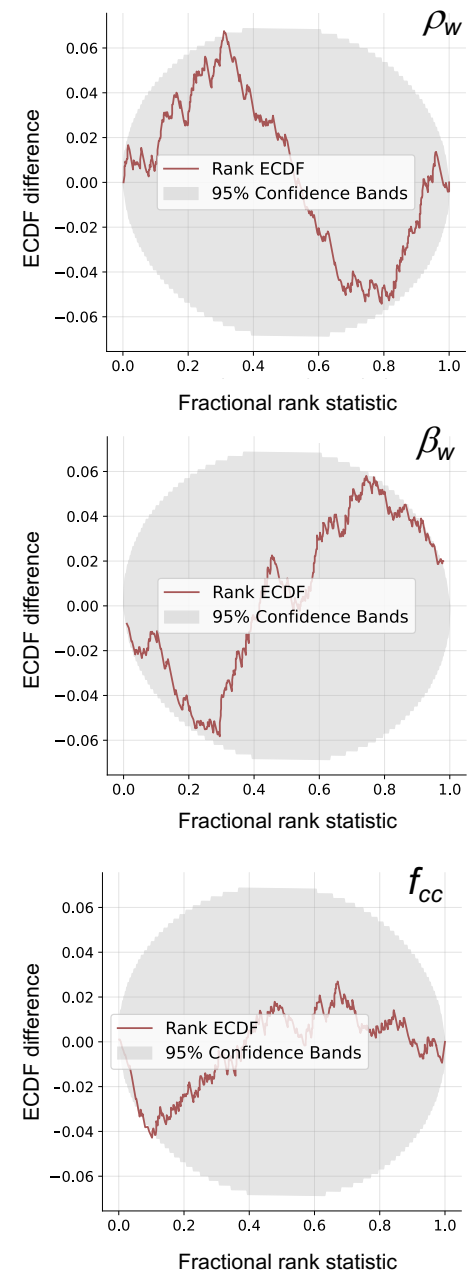**B**

Prior distribution

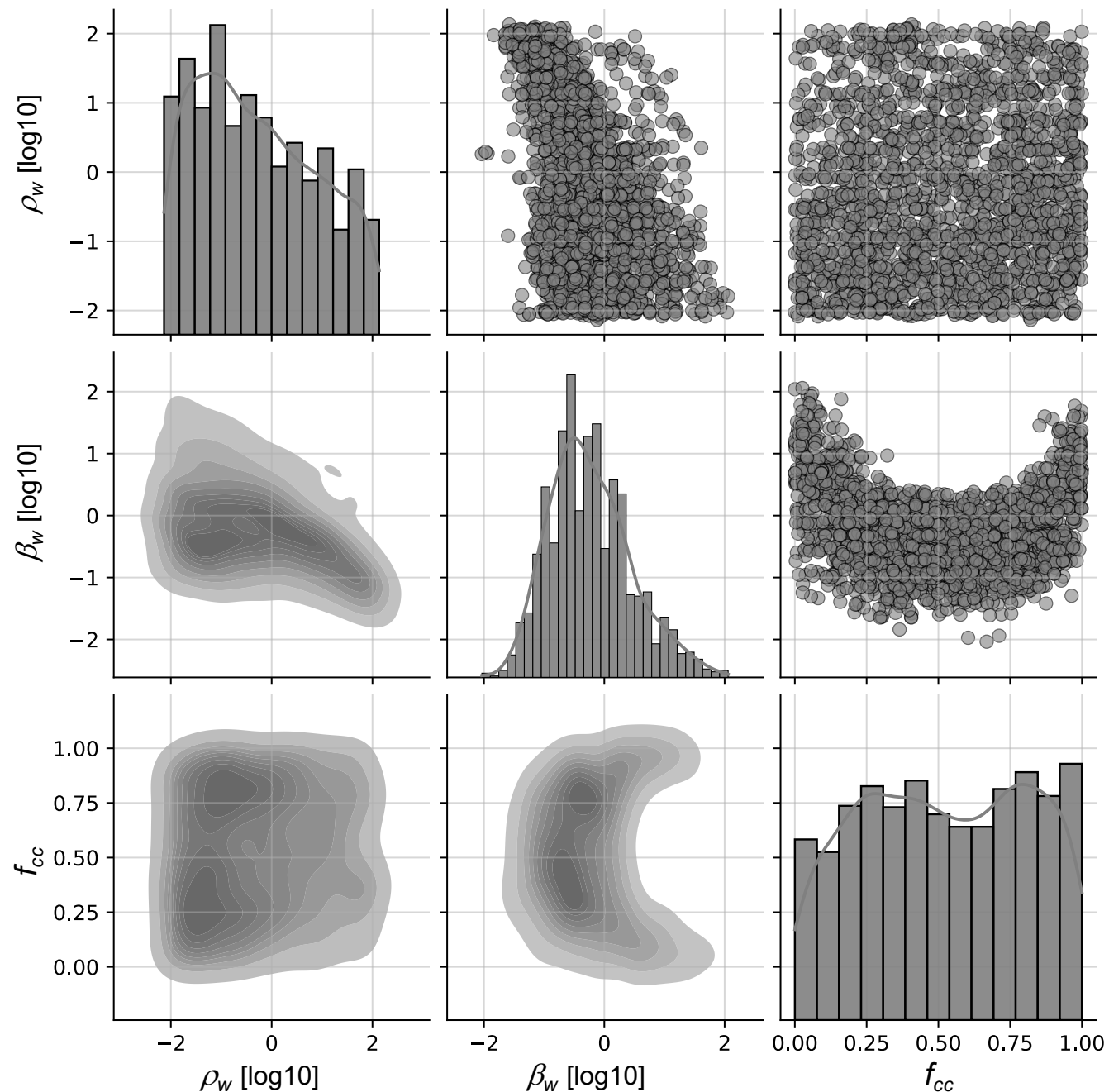

Supplement: S5 Fig — (A) Empirical cumulative distribution functions (ECDF) and (B) prior distribution for the estimated parameters of model MHOM. (PDF) [file pcbi.1014248.s006.pdf]

**A** $M_{\text{HAE}}$ 

w/o. spatial

with spatial

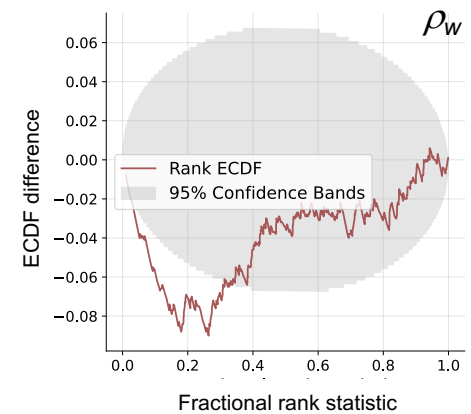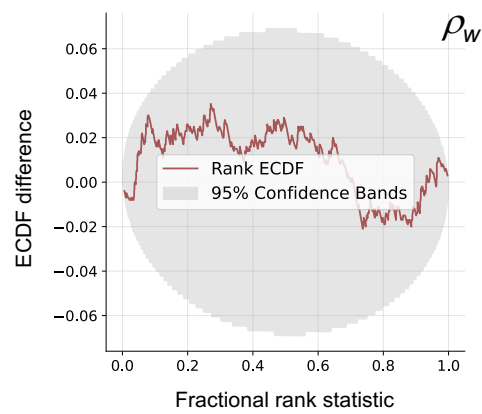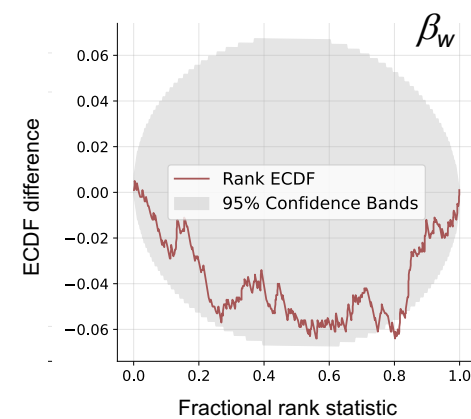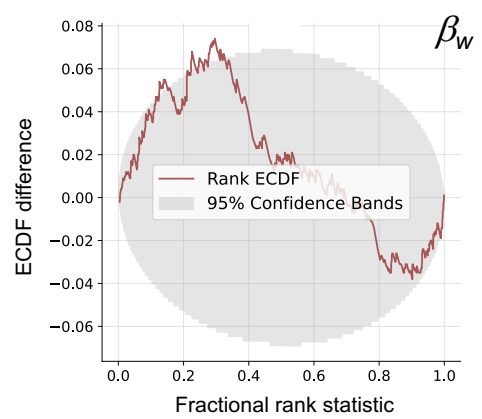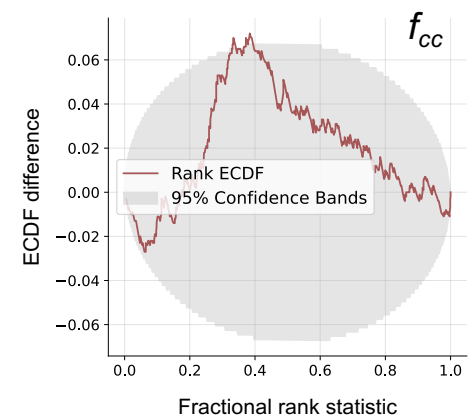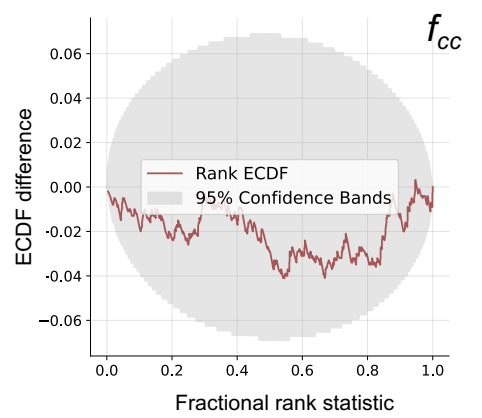**B**

Prior distribution

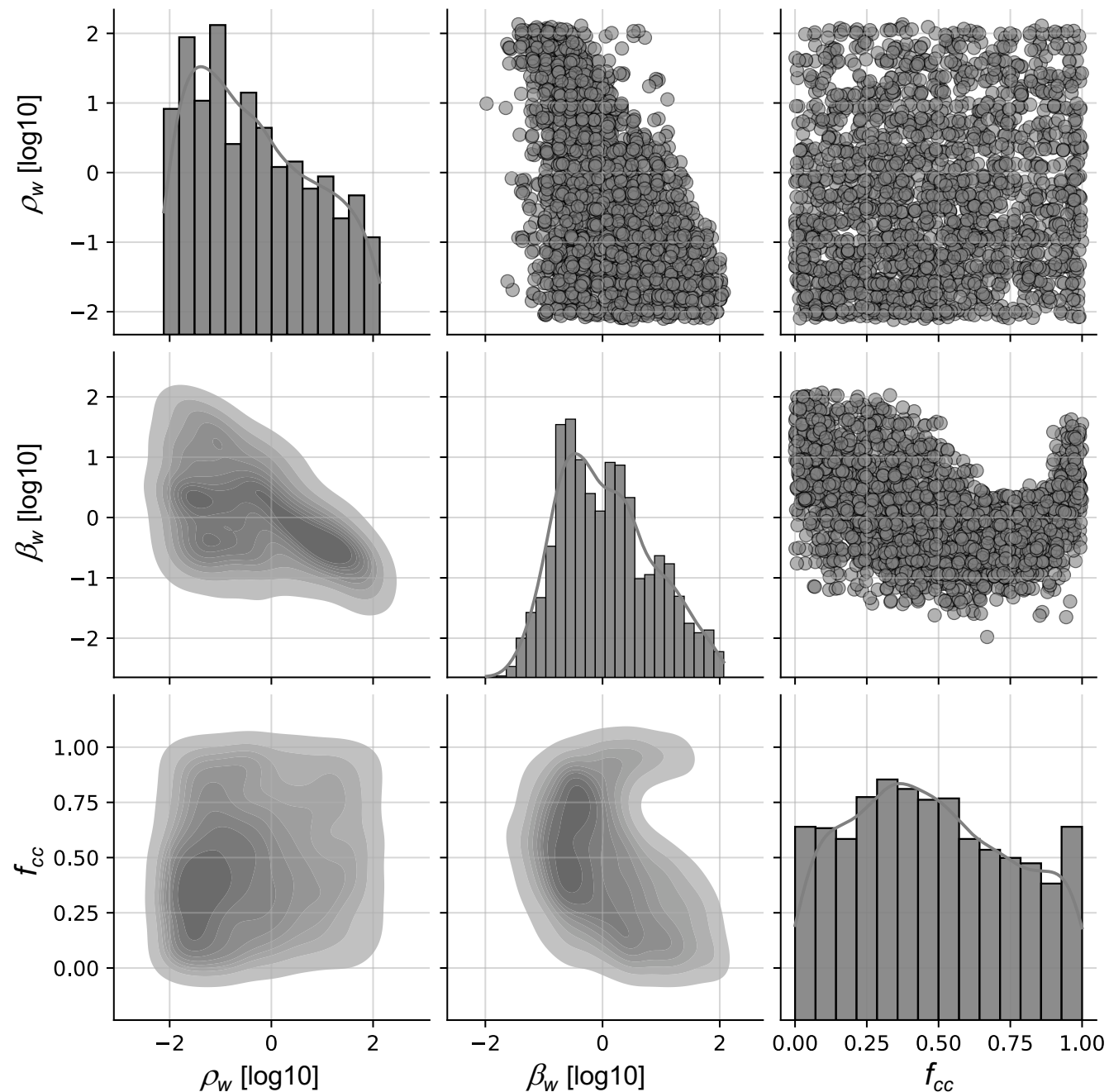

Supplement: S6 Fig — (A) Empirical cumulative distribution functions (ECDF) and (B) prior distribution for the estimated parameters of model MHAE. (PDF) [file pcbi.1014248.s007.pdf]

**A**

viral kinetics

 $M_{\text{HAE-}\Phi}$ 

innate immunity

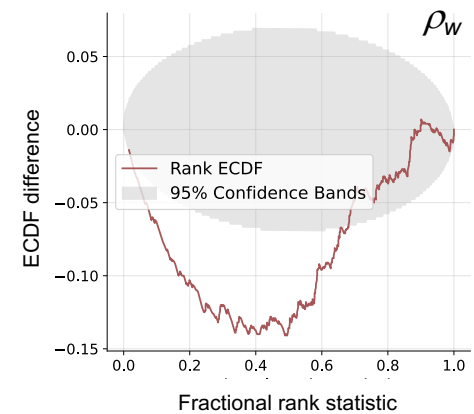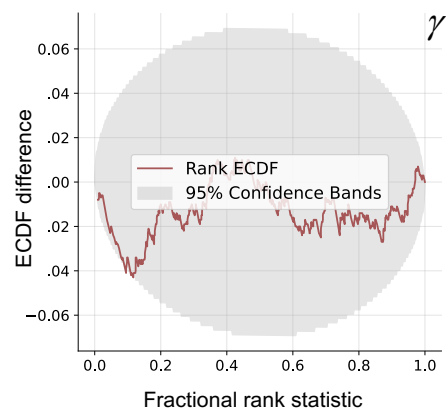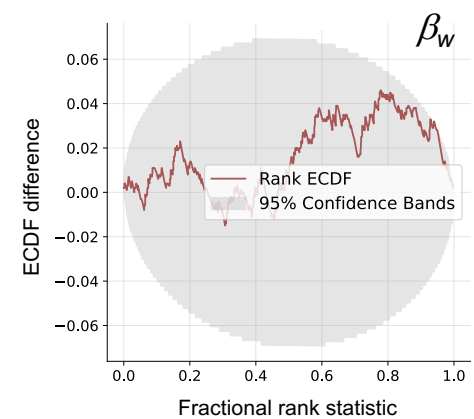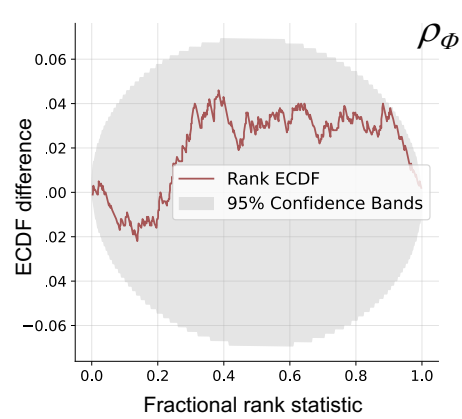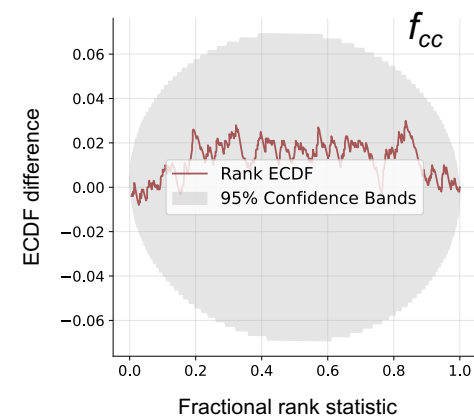**B**

Prior distribution

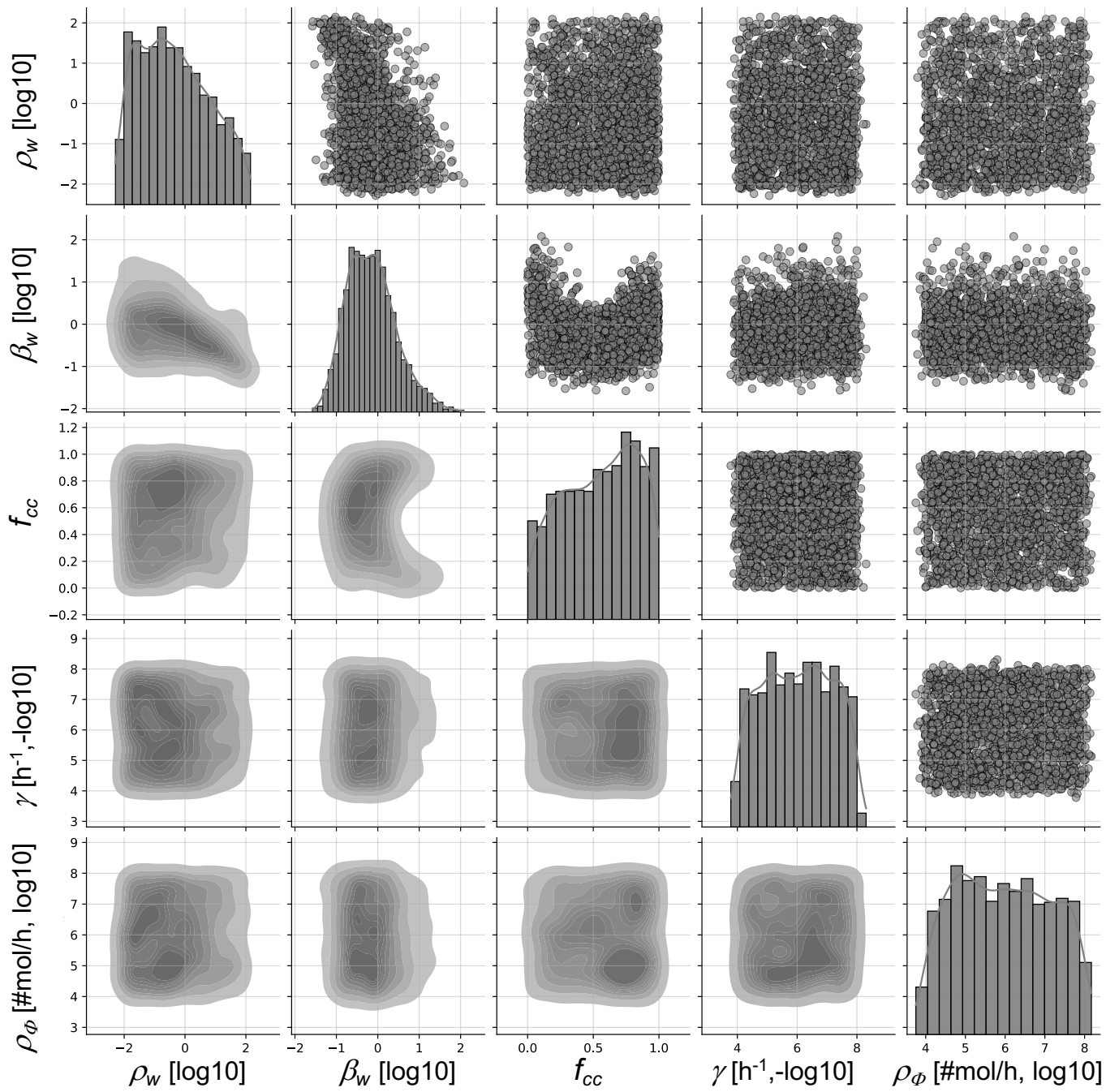

Supplement: S7 Fig — (A) Empirical cumulative distribution functions (ECDF) and (B) prior distribution for the estimated parameters of model MHAE−Φ. (PDF) [file pcbi.1014248.s008.pdf]

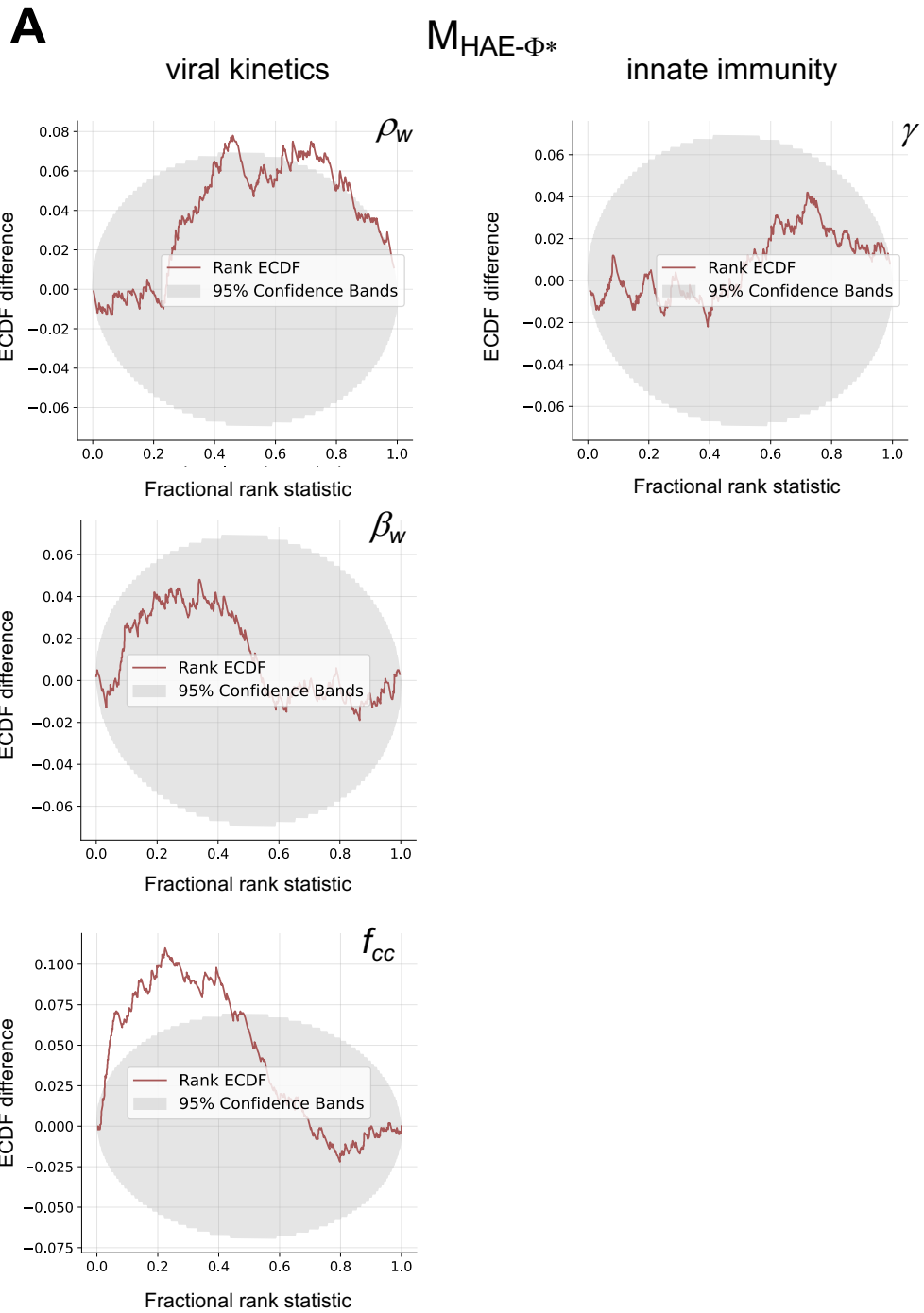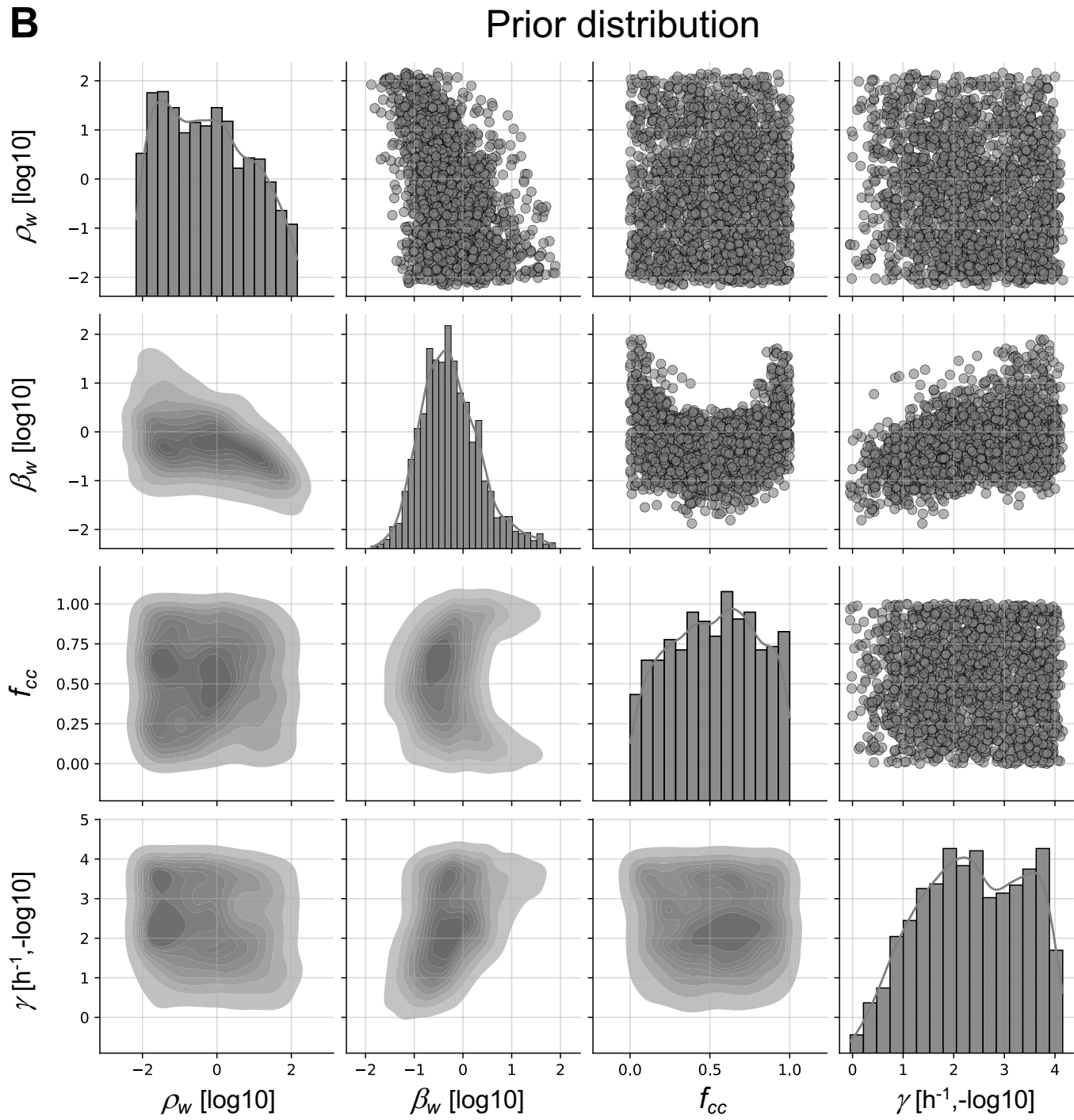

Supplement: S8 Fig — (A) Empirical cumulative distribution functions (ECDF) and (B) prior distribution for the estimated parameters of model MHAE−Φ⋆. (PDF) [file pcbi.1014248.s009.pdf]
