# Supplementary material for: Multimodal data integration to determine viral and innate immune kinetics in human airway epithelium
Source: PLoS Comput Biol. 2026 May 20;22(5):e1014248. doi: 10.1371/journal.pcbi.1014248 (PMC13245872; doi:10.1371/journal.pcbi.1014248)
Supplement: S1 Table — General parameters for the cellular Potts model (CPM) describing human airway epithelium. (PDF) [file pcbi.1014248.s010.pdf]

S1 Table: CPM parameters. General parameters for the cellular Potts model describing human airway epithelium. (*a.u. - arbitrary units*)

**Description**	**Parameter**	**Unit**	**Value**		
Total size	A	$\mu m \times \mu m$	491.47×491.47		
Total cell number	N		10^4		
Simulation step size		h	0.25		
Effectiveness of cell-free transmission	w_{cf}		N/15		
Effectiveness of cell-to-cell transmission	w_{cc}		1/20		
Initial viral load	V_0	Virions per μm^2	1.8×10^3		
**Celltype specific parameters**					
			**Basal**	**Secretory**	**Ciliated**
M_{HOM}					
Target volume	A_T	μm^2	-	-	60
Volume strength	V_S	*a.u.*	-	-	100
Surface constraint	Ψ	*a.u.*	-	-	100
Cell-to-cell transmission, relative infection parameter	β_{CC}		-	-	4.33
Cell-to-cell transmission, relative infecting parameter	ρ_{CC}		-	-	0.4859
$M_{HAE} + M_{HAE-\phi} + M_{HAE-\phi*}$					
Target volume	A_T	μm^2	60	60	60
Volume strength	V_S	*a.u.*	10	100	100
Surface constraint	Ψ	*a.u.*	0.9	0.9	0.9
Cell-to-cell transmission, relative infection parameter	β_{CC}		1	1.536	4.33
Cell-to-cell transmission, relative infecting parameter	ρ_{CC}		1	0.5786	0.4859
