# Supplementary material for: Multimodal data integration to determine viral and innate immune kinetics in human airway epithelium
Source: PLoS Comput Biol. 2026 May 20;22(5):e1014248. doi: 10.1371/journal.pcbi.1014248 (PMC13245872; doi:10.1371/journal.pcbi.1014248)
Supplement: S2 Table — Parameters used within the different model systems to describe viral, immune and tissue regeneration dynamics. (PDF) [file pcbi.1014248.s011.pdf]

S2 Table: Dynamic parameters. Parameters used within the different model systems to describe viral, immune and tissue regeneration dynamics.

Description	Parameter	Unit	Value	Ref.
$M_{HOM} + M_{HAE} + M_{HAE-\phi} + M_{HAE-\phi*}$				
Viral clearance	c_V	h^{-1}	0.63	[1,2]
Viral diffusion rate	D_V	$\mu m^2 h^{-1}$	60	[3,4]
Half-saturation constant of the IFN induced inhibition of viral production	IC_{50}	$\log_{10}$	3.70	[5]
Hill coefficient of the IFN induced inhibition of viral production	ν		2	[5]
Relative contribution of cell-to-cell transmission	f_{CC}		estimated	
Weighting factor of viral transmission rate	β_w	$\log_{10}$	estimated	
Weighting factor of viral production rate	ρ_w	$\log_{10}$	estimated	
M_{HOM}				
Loss rate of susceptible cells	δ_S	$10^{-2}d^{-1}$	2.86	[3]
Death rate of infectious cells	δ_J	$10^{-2}d^{-1}$	7.43	[3]
Viral transmission rate	β	$-\log_{10} h^{-1}$	9.43	[3]
Viral transmission rate (cell-to-cell)	β_{CC}		4.33	[3]
Rate of infected cell to turn productively infected	κ	h^{-1}	0.22	[3]
Viral production rate	ρ	$\log_{10}$ cp	4.69	[3]
$M_{HAE} + M_{HAE-\phi} + M_{HAE-\phi*}$				
Maximal proliferation rate of basal cells	α	d^{-1}	0.34	[3]
Differentiation rate basal-secretory	λ_S	d^{-1}	8.41	[3]
Differentiation rate secretory-ciliated	λ_C	d^{-1}	16.23	[3]
Loss rate of ciliated cells	δ_C	$10^{-2}d^{-1}$	2.86	[3]
Loss rate of secretory cells	δ_S	$10^{-2}d^{-1}$	8.28	[3]
Loss rate of basal cells	δ_B	$10^{-2}d^{-1}$	1.39	[3]
Death rate of infectious cells (ciliated)	$\delta_{J,C}$	$10^{-2}d^{-1}$	7.43	[3]
Death rate of infectious cells (secretory)	$\delta_{J,S}$	$10^{-2}d^{-1}$	7.7	[3]
Death rate of infectious cells (basal)	$\delta_{J,B}$	$10^{-2}d^{-1}$	8.09	[3]
Viral transmission rate (ciliated)	β_C	$-\log_{10} h^{-1}$	9.43	[3]
Viral transmission rate (secretory)	β_S	$-\log_{10} h^{-1}$	9.4	[3]
Viral transmission rate (basal)	β_B	$-\log_{10} h^{-1}$	9.36	[3]
Viral transmission rate (cell-to-cell, ciliated)	$\beta_{CC,C}$		4.33	[3]
Viral transmission rate (cell-to-cell, secretory)	$\beta_{CC,S}$		1.54	[3]
Viral transmission rate (cell-to-cell, basal)	$\beta_{CC,B}$		1	[3]
Rate of infected cell to turn productively infected (ciliated)	κ_C	h^{-1}	0.22	[3]
Rate of infected cell to turn productively infected (secretory)	κ_S	h^{-1}	0.18	[3]
Rate of infected cell to turn productively infected (basal)	κ_B	h^{-1}	0.19	[3]
Viral production rate (ciliated)	ρ_C	$\log_{10}$ cp	4.69	[3]
Viral production rate (secretory)	ρ_S	$\log_{10}$ cp	4.61	[3]
Viral production rate (basal)	ρ_B	$\log_{10}$ cp	4.38	[3]
$M_{HAE-\phi} + M_{HAE-\phi*}$				
Interferon degradation	c_ϕ	h^{-1}	0.1	[6]
Interferon diffusion rate	D_ϕ	$\mu m^2 h^{-1}$	600	[6,7]
Interferon production rate (ciliated, secretory, basal)	ρ_ϕ	$\log_{10}$ mol$/h$	estimated	
Protection rate (ciliated, secretory, basal)	γ	$-\log_{10} h^{-1}$	estimated	
Rate of refractory cells to susceptible (ciliated, secretory, basal)	λ_R	h^{-1}	0.04	[6]
Rate of infectious cells to interferon production (ciliated, secretory, basal)	κ_ϕ	h^{-1}	0.02	[6]
$M_{HAE-\phi*}$				
Interferon production rate (ciliated, secretory, basal)	ρ_ϕ	$\log_{10}$ mol$/h$	4.56	[8]

References

1. Goyal A, Cardozo-Ojeda EF, Schiffer JT. Potency and timing of antiviral therapy as determinants of duration of SARS-CoV-2 shedding and intensity of inflammatory response. Science Advances. 2020;6(47):eabc7112. doi:10.1126/sciadv.abc7112.

2. Stanifer ML, Kee C, Cortese M, Zumaran CM, Triana S, Mukenhirn M, et al. Critical Role of Type III Interferon in Controlling SARS-CoV-2 Infection in Human Intestinal Epithelial Cells. Cell Reports. 2020;32(1):107863. doi:10.1016/j.celrep.2020.107863.

3. Raach B, Bundgaard N, Haase MJ, Starruß J, Sotillo R, Stanifer ML, et al. Influence of cell type specific infectivity and tissue composition on SARS-CoV-2 infection dynamics within human airway epithelium. PLoS Comput Biol. 2023;19(8):e1011356. doi:10.1371/journal.pcbi.1011356.

4. Durso-Cain K, Kumberger P, Schälte Y, Fink T, Dahari H, Hasenauer J, et al. HCV Spread Kinetics Reveal Varying Contributions of Transmission Modes to Infection Dynamics. Viruses. 2021;13(7):1308. doi:10.3390/v13071308.

5. Talemi SR, Bartenschlager M, Schmid B, Ruggieri A, Bartenschlager R, Höfer T. Dengue virus is sensitive to inhibition prior to productive replication. Cell Reports. 2021;37(2):109801. doi:10.1016/j.celrep.2021.109801.

6. Schmid B, Rinas M, Ruggieri A, Acosta EG, Bartenschlager M, Reuter A, et al. Live Cell Analysis and Mathematical Modeling Identify Determinants of Attenuation of Dengue Virus 2'-O-Methylation Mutant. PLOS Pathogens. 2015;11(12):e1005345. doi:10.1371/journal.ppat.1005345.

7. Hu J, Nudelman G, Shimoni Y, Kumar M, Ding Y, López C, et al. Role of cell-to-cell variability in activating a positive feedback antiviral response in human dendritic cells. PloS One. 2011;6(2):e16614. doi:10.1371/journal.pone.0016614.

8. Han Q, Bradshaw EM, Nilsson B, Hafler DA, Love JC. Multidimensional analysis of the frequencies and rates of cytokine secretion from single cells by quantitative microengraving. Lab on a Chip. 2010;10(11):1391–1400. doi:10.1039/b926849a.
