# Supplementary material for: Multimodal data integration to determine viral and innate immune kinetics in human airway epithelium
Source: PLoS Comput Biol. 2026 May 20;22(5):e1014248. doi: 10.1371/journal.pcbi.1014248 (PMC13245872; doi:10.1371/journal.pcbi.1014248)
Supplement: S3 Table — Posterior estimates for the parameters describing viral and immune dynamics for the experimental SARS-CoV-2 HAE-culture systems using model MHAE−Φ⋆ adapted to the experimental protocol. (PDF) [file pcbi.1014248.s012.pdf]

S3 Table: Posterior estimates for SARS-CoV-2 spread in HAE culture systems. Posterior estimates for the parameters describing viral and immune dynamics for the experimental SARS-CoV-2 HAE-culture systems using model $M_{HAE-\Phi\star}$ adapted to the experimental protocol.

Description	Parameter	Unit	Value	95% CrI
Scaling relative contribution of cell-to-cell transmission	f_{cc}		0.61	[0.23, 0.96]
Scaling factor of viral transmission rate	β_w	$-\log_{10}$	0.90	[0.04, 1.28]
Scaling factor of viral production rate	ρ_w	$\log_{10}$	1.68	[1.19, 2.06]
Protection rate (ciliated, secretory, basal)	γ	$-\log_{10} h^{-1}$	2.47	[1.05, 3.71]
