# Supplementary material for: Multimodal data integration to determine viral and innate immune kinetics in human airway epithelium
Source: PLoS Comput Biol. 2026 May 20;22(5):e1014248. doi: 10.1371/journal.pcbi.1014248 (PMC13245872; doi:10.1371/journal.pcbi.1014248)
Supplement: S4 Table — Optimal hyper-parameters identified for each of the different models and training data considered. (PDF) [file pcbi.1014248.s013.pdf]

S4 Table: Optimal hyperparameters for neural network architecture and training. Optimal hyper-parameters identified for each of the different models and training data considered.

**Simulator**	**Type of Training Data**	**Summary Dimensions**	**Coupling Layers**	**Dropout Rate**	**Learning Rate**	**Number of Epochs**
Synthetic data / Validation of approach						
M_{HOM}	Bulk	6	6	0.1	0.0005	46
M_{HOM}	Bulk + Image	6	6	0.1	0.0005	44
M_{HAE}	Bulk	6	6	0.05	0.0005	24
M_{HAE}	Bulk + Image	10	6	0	0.0005	26
$M_{HAE-\Phi}$	Bulk + Image	10	6	0.05	0.0005	46
$M_{HAE-\Phi*}$	Bulk + Image	10	4	0.05	0.0005	52
Application to experimental data						
$M_{HAE-\Phi*}$	Bulk + Image	22	4	0.1	0.0001	150
